# Integrated drug resistance and leukemic stemness gene-expression scores predict outcomes in large cohort of over 3500 AML patients from 10 trials

**DOI:** 10.1038/s41698-024-00643-5

**Published:** 2024-08-01

**Authors:** Abdelrahman H. Elsayed, Xueyuan Cao, Richard J. Marrero, Nam H. K. Nguyen, Huiyun Wu, Yonhui Ni, Raul C. Ribeiro, Herold Tobias, Peter J. Valk, François Béliveau, Guillaume Richard-Carpentier, Josée Hébert, C. Michel Zwaan, Alan Gamis, Edward Anders Kolb, Richard Aplenc, Todd A. Alonzo, Soheil Meshinchi, Jeffrey Rubnitz, Stanley Pounds, Jatinder K. Lamba

**Affiliations:** 1 Department of Pharmacotherapy and Translational Research, College of Pharmacy, University of Florida, Gainesville, FL USA; 2 Department of Health Promotion and Disease Prevention, University of Tennessee Health Science Center, Memphis, TN USA; 3 Department of Biostatistics, St. Jude Children’s Research Hospital, Memphis, TN USA; 4 Department of Oncology, St. Jude Children’s Research Hospital, Memphis, TN USA; 5 Department of Medicine III, Ludwig Maximillans University Hospital, LMU Munich, Germany; 6 Department of Hematology, Erasmus Medical Center Cancer Institute, University Medical Center Rotterdam, Rotterdam, The Netherlands; 7 Quebec leukemia cell bank, Hôpital Maisonneuve-Rosemont, Montréal, QC Canada; 8 Princess Margaret Cancer Centre, University Health Network, Toronto, ON Canada; 9 Department of Medicine, Division of Medical Oncology and Hematology, Temerty Faculty of Medicine, University of Toronto, Toronto, ON Canada; 10 Division of Hematology and Oncology, Hôpital Maisonneuve-Rosemont, Montréal, QC Canada; 11 Department of Medicine, Faculty of Medicine, Université de Montréal, Montréal, QC Canada; 12 Princess Máxima Center for Pediatric Oncology, Utrecht, the Netherlands; 13 Department of Pediatric Oncology, Erasmus MC-Sophia Children’s Hospital, Rotterdam, the Netherlands; 14 Division of Hematology/Oncology, Children’s Mercy Kansas City, Kansas City, MO USA; 15 Nemours Center for Cancer and Blood Disorders, Alfred I. DuPont Hospital for Children, Wilmington, DE USA; 16 Department of Pediatrics, Perelman School of Medicine, University of Pennsylvania, Philadelphia, PA USA; 17 COG Statistics and Data Center, Monrovia, CA USA; 18 Department of Population and Public Health Sciences, Keck School of Medicine, University of Southern California, Los Angeles, CA USA; 19 Clinical Research Division, Fred Hutchinson Cancer Research Center, Seattle, WA USA; 20 University of Florida Health Cancer Center, University of Florida, Gainesville, FL USA

**Keywords:** Predictive markers, Acute myeloid leukaemia

## Abstract

In this study, we leveraged machine-learning tools by evaluating expression of genes of pharmacological relevance to standard-AML chemotherapy (ara-C/daunorubicin/etoposide) in a discovery-cohort of pediatric AML patients (*N* = 163; NCT00136084) and defined a 5-gene-drug resistance score (ADE-RS5) that was predictive of outcome (high MRD1 positivity *p* = 0.013; lower EFS *p* < 0.0001 and OS *p* < 0.0001). ADE-RS5 was integrated with a previously defined leukemic-stemness signature (pLSC6) to classify patients into four groups. ADE-RS5, pLSC6 and integrated-score was evaluated for association with outcome in one of the largest assembly of ~3600 AML patients from 10 independent cohorts (1861 pediatric and 1773 adult AML). Patients with high ADE-RS5 had poor outcome in validation cohorts and the previously reported pLSC6 maintained strong significant association in all validation cohorts. For pLSC6/ADE-RS5-integrated-score analysis, using Group-1 (low-scores for ADE-RS5 and pLSC6) as reference, Group-4 (high-scores for ADE-RS5 and pLSC6) showed worst outcome (EFS: *p* < 0.0001 and OS: *p* < 0.0001). Groups-2/3 (one high and one low-score) showed intermediate outcome (*p* < 0.001). Integrated score groups remained an independent predictor of outcome in multivariable-analysis after adjusting for established prognostic factors (EFS: Group 2 vs. 1, HR = 4.68, *p* < 0.001, Group 3 vs. 1, HR = 3.22, *p* = 0.01, and Group 4 vs. 1, HR = 7.26, *p* < 0.001). These results highlight the significant prognostic value of transcriptomics-based scores capturing disease aggressiveness through pLSC6 and drug resistance via ADE-RS5. The pLSC6 stemness score is a significant predictor of outcome and associates with high-risk group features, the ADE-RS5 drug resistance score adds further value, reflecting the clinical utility of simultaneous testing of both for optimizing treatment strategies.

## Introduction

Standard induction treatment of patients with AML consists of cytarabine (ara-C), daunorubicin with or without etoposide (ADE or DA standard chemotherapy)^1,2^. Despite major advances in AML treatment, the development of drug resistance is one of the major causes of treatment failure and relapse in AML patients^1–3^. Previous studies have shown that genes involved in the metabolism or targeted by ADE chemotherapeutic agents (PK/PD genes) associate with the development of drug resistance and poor outcomes; however, these genes have been predominantly studied in isolation^3–5^. Because of concomitant administration of these drugs as induction regimen, we reasoned that comprehensive and systematic transcriptomic evaluation of genes of pharmacological significance to ara-C, daunorubicin and etoposide will help in providing a drug resistance score predictive of treatment outcomes in AML patients. To fulfill this goal, we cataloged a list of 67 genes involved in the metabolism or transport of ara-C, daunorubicin or etoposide and their potential drug targets. These genes can contribute to the emergence of drug resistance through various mechanisms as: (1) reduced cellular uptake due to low levels of uptake transporters; (2) increased efflux due to high expression of efflux transporters; (3) decreased expression or activity of enzymes responsible for the activation of pro-drugs; (4) increased expression or activity of enzymes responsible for the drug inactivation; (5) alterations in the expression or function of the molecular targets of the drugs. These key players have been well-established in impacting drug pharmacokinetics or pharmacodynamics a comprehensive transcriptomic evaluation using machine learning tools to develop a drug resistance signature has not been done. However, comprehensive evaluation of transcriptomic of these players have not been performed in AML. Previously, Least Absolute Shrinkage and Selection Operator (known as LASSO) based regression analysis defined a leukemic stemness score consisting of gene expression levels of 17 genes that was predictive of outcome has been reported^6^. A follow-up work defined a pediatric leukemic stemness score consisting of 6 genes in AML^7^. Within ALL, lasso analysis has been utilized to define prognostic risk factors^8^.

In this study, we evaluated the transcriptome of 67 pharmacologically relevant genes (listed in Table S1) in pediatric AML patients treated on the AML02 multi-center clinical trial. We utilized LASSO penalized regression on clinical outcome data to examine the significance of these genes and developed an ADE-Resistance Score (ADE-RS5) that was further validated in 10 independent AML cohorts. Recently our group developed a six-gene leukemic stem cell (pLSC6) score that associated with risk-groups and patient outcomes in pediatric AML^7^. Further combining the pLSC6 and ADE-RS5 score groups to incorporate both disease aggressiveness, as implied by the stemness score, and drug resistance, as reflected by resistance score was conducted across 10 cohorts of pediatric and adult AML patients, totaling 3634 individuals.

## Results

### Expression of five pharmacological genes defines a drug resistance score of prognostic value in AML02 discovery cohort

**Fig. 1:**
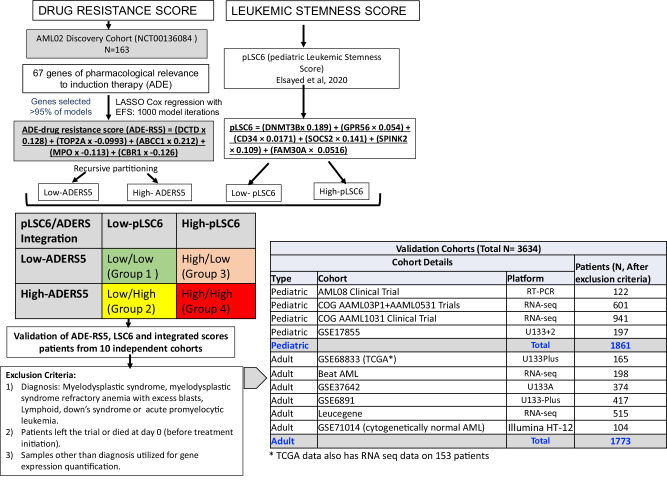
Overall study design.

LASSO penalized Cox regression model using mRNA expression levels of 67 genes with EFS in 163 patients (model-development cohort) treated on multi-site AML02 trial identified five genes that passed at least 950 of 1000 leave-10%-out cross-validation replications of this analysis (Fig. 1 and Supplementary Fig. 1). This rigorous model-development process defined a five-gene ADE-Resistance Score (ADE-RS5) which was computed for each patient using gene expression weighted by the regression coefficients as defined in the following equation:

1 $$\begin{array}{l}{\rm{ADES}}-{\rm{RS}}5=\left({DCTD}^* 0.128\right)+\left({TOP}2A^* -0.0993\right)+\left({ABCC}1^* 0.212\right)\\\qquad\qquad\qquad\quad+\left({MPO}^* -0.113\right)+\left({CBR}1^* -0.126\right)\end{array}$$

**Fig. 2:**
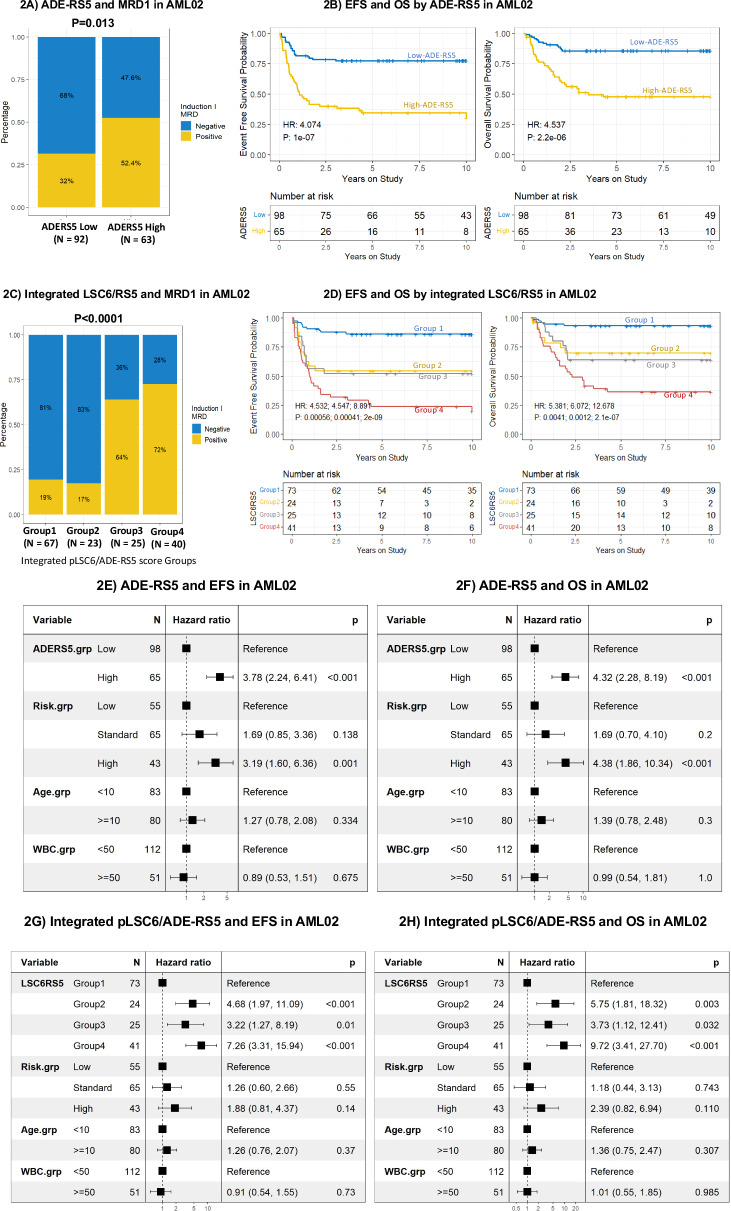
ADE-resistance score predicts AML outcome in discovery cohort. High ADE-resistance score (ADE-RS5) significantly predicts MRD1 positivity (**A**), lower EFS and OS (**B**) probability in AML02 discovery cohort (*n* = 163). Association of the four groups classification based on integration of pLSC6 and ADE-RS5 scores (LSC6/RS5) with MRD1 (**C**), EFS and OS (**D**) probability in AML02 cohort. Forest plot showing results of multivariable cox regression analysis of association of ADE-RS, and the integrated pLSC6/ADE-RS5 score groups with EFS (**E** and **G**) and OS (**F** and **H**) after adjusting for risk group assignment, diagnostic WBC count, FLT3 status and age. For integrated LSC6RS5 scores: Group 1 = both LSC6 and ADE-RS5 scores are low; Group 2 = Low LSC6 score and High ADE-RS5 score; Group 3 = High LSC6 score and low ADE-RS5 score; Group 4= both LSC6 and ADE-RS5 scores are high.

Each unit increase in ADE-RS5 was associated with a 7.32-fold increase in the rate of EFS events (*p* < 0.00001, 95% CI = 3.75–14.28) in a simple single-predictor Cox regression model. Dichotomization by recursive-partitioning resulted in classification of patients into two groups: low ADE-RS5 (*n* = 98 patients, 60%) or high ADE-RS5 score group (*n* = 65 patients; 40%). Though ADE-RS5 score groups did not differ by age, gender, race, risk group, FLT3-ITD status or WBC count at diagnosis, difference in distribution of cytogenetics was observed, as shown in Supplementary Table 2 summarizing patients characteristics by score groups. Within the discovery cohort, high ADE-RS5 score was a significant predictor of higher MRD1 positivity (OR = 2.39, 95% CI = 1.23–4.63, *p* = 0.013 Fig. 2A), lower EFS (HR = 4.07, 95% CI = 2.43–6.84; *p* < 0.0001 Fig. 2B), and OS probability in AML02 cohort (HR = 4.54, 95% CI = 2.42-8.49; *p* < 0.0001, Fig. 2B).

### Integration of ADE-RS5 score with previously established pLSC6 score in AML02 discovery cohort

We previously developed a clinically significant leukemic stemness score in pediatric AML and designated it as pLSC6 (derived from expression levels of *DNMT3B, GPR56, CD34, SOCS2, SPINK2*, and *FAM30A)*. Patients within low pLSC6 score group previously showed better outcome as compared high pLSC6 group^7^. ADE-RS5 was tested within the pLSC6 score groups. Within low-pLSC6 score group (*N* = 97), high ADE-RS5 score was significantly associated with lower EFS (HR = 4.25; 95%CI = 1.08–10.04, *P* = 0.0009; Supplementary Fig. 2A) and OS (HR = 4.96; 95%CI = 1.57–15.64, *P* = 0.0063; Supplementary Fig. 2B) compared to the group of patients with low ADE-RS5 score. Similarly, within the high-pLSC6 score group (*N* = 66), patients with high ADE-RS5 score experienced lower EFS (HR = 1.98; 95%CI = 1.02–3.86, *P* = 0.044; Supplementary Fig. 2A), and OS (HR = 2.12; 95%CI = 0.99-4.52, *P* = 0.053; Supplementary Fig. 2B) as compared to low ADE-RS5 group. Based on these results, ADE-RS5 further enhanced the prognostic value of pLSC6 in predicting poor outcomes in patients with AML and thus we integrated pLSC6 and ADE-RS5 scores to classify patients into four groups (Fig. 1):

**Group-1: Low**: patients with low pLSC6 and low ADE-RS5 scores

**Group-2: Low/High**: patients with low pLSC6 and high ADE-RS5 scores

**Group-3: High/Low**: patients with high pLSC6 and low ADE-RS5 scores

**Group-4: High**: patients with high pLSC6 and high ADE-RS5 scores

Patient characteristics by the four-group assignment for the discovery cohort (AML02 trial) is summarized in Supplementary Table 2 and initial risk group assignment, cytogenetic features and FLT3-ITD status were the diagnostic features that differed by the groups. Patients in the integrated Group 4 (both pLSC6 and ADE-RS5 high scores) and Group 3 (high pLSC6 and low ADE-RS5) experienced greater MRD1 positivity (72% and 64%, Fig. 2C) as compared to Groups 1 and 2 (19% and 17%) implying it might be driven by high pLSC6 score.

With respect to survival outcomes patients within Group 4 had lower EFS (HR = 8.89, *p* < 0.0001) and OS (HR = 12.68, *p* < 0.0001) as compared to patients in Group 1 (Fig. 2D). Patients within Groups 2 and 3 showed intermediate outcome with significantly poor outcome as compared to the Group 1 (all *p* < 0.005, Fig. 2D).

In multivariable analysis after adjusting for diagnostic risk group assignment, WBC count, FLT3 status and age, ADE-RS5 remained an independent predictor of lower EFS and OS, (EFS: HR = 3.78, 95%CI = 2.24–6.41, *p* < 0.001; OS: HR = 4.32, 95%CI = 2.28-8.2, *P* < 0.001; Fig. 2E, F). Furthermore, in an integrated score group analysis with Group 1 as a reference group, significant poor outcome was observed in Groups 2, 3 and 4 for EFS (Group 2 vs. 1), HR = 4.68, *p* < 0.001, Group 3 vs. 1, HR = 3.22, *p* = 0.01, and Group 4 vs. 1, HR = 7.26, *p* < 0.001 Fig. 2G and OS (Group 2 vs. 1, HR = 5.75, *p* = 0.003, Group 3 vs. 1, HR = 3.73, p = 0.032, and Group 4 vs. 1, HR = 9.72, p < 0.001, Fig. 2H) in AML02 cohort.

### Validation of transcriptomic based prognostic scores in >3000 patients from independent pediatric and adult clinical trials

We performed analysis of pLSC6, ADE-RS5 and integrated scores by combining all the pediatric cohorts together (4 different trials, total *n* = 1861) and all the adult cohorts together (5 different trials, total *n* = 1669). Distribution of patient characteristics by pLSC6, ADE-RS5 and integrated-pLSC6/ADE-RS5 scores across pediatric and adult validation cohorts is provided in Table 1. Overall consistent with our previous report, pLSC6 score group was significantly associated with patient’s risk group assignment, cytogenetics and FLT3 status and in addition to these factors, ADE-RS5 was associated with age in the combined pediatric and gender in the combined adult cohort.

**Table 1:** Distribution of patient characteristics in combined pediatric and adult AML validation cohorts (*N* = 1861 pediatric and 1669 adult AML patients)

		pLSC6 Score Groups			ADE-RS5 Score Groups			Integrated LSC6/ADE-RS5 Four Score Groups							
Variables	Level	Low	High	*P* value	Low	High	*P* value	Group 1	Group 2	Group 3		Group 4		P value	
Pediatric AML Validation Cohort: Total *N* = 1861															
Gender	Female	539 (48.3)	337 (45.2)	0.1791	520 (46.6)	356 (47.7)	0.6721	385 (49)	154 (46.7)		135 (40.9)		202 (48.6)		0.0828
	Male	576 (51.7)	409 (54.8)		595 (53.4)	390 (52.3)		400 (51)	176 (53.3)		195 (59.1)		214 (51.4)		
Age group	<10	564 (50.6)	384 (51.5)	0.7348	535 (48)	413 (55.4)	**0.002**	375 (47.8)	189 (57.3)		160 (48.5)		224 (53.8)		**0.0146**
	>=10	551 (49.4)	362 (48.5)		580 (52)	333 (44.6)		410 (52.2)	141 (42.7)		170 (51.5)		192 (46.2)		
Race	White	708 (77.7)	496 (79.5)	0.3103	726 (78.4)	478 (78.5)	0.9903	497 (77.4)	211 (78.4)		229 (80.6)		267 (78.5)		0.4569
	Black	119 (13.1)	84 (13.5)		122 (13.2)	81 (13.3)		89 (13.9)	30 (11.2)		33 (11.6)		51 (15)		
	Other	84 (9.2)	44 (7.1)		78 (8.4)	50 (8.2)		56 (8.7)	28 (10.4)		22 (7.7)		22 (6.5)		
Risk group	Low	594 (54.6)	113 (15.4)	**<0.0001**	549 (50.1)	158 (21.8)	**<0.0001**	480 (62.5)	114 (35.6)		69 (21.1)		44 (10.9)		**<0.0001**
	Standard	443 (40.7)	484 (66.1)		475 (43.4)	452 (62.3)		262 (34.1)	181 (56.6)		213 (65.1)		271 (66.9)		
	High	51 (4.7)	135 (18.4)		71 (6.5)	115 (15.9)		26 (3.4)	25 (7.8)		45 (13.8)		90 (22.2)		
Cytogenetic group	t(8;21)	268 (24.7)	0 (0)	**<0.0001**	198 (18.1)	70 (9.7)	**<0.0001**	198 (25.8)	70 (22)		0 (0)		0 (0)		**<0.0001**
	inv(16)	204 (18.8)	21 (2.9)		209 (19.1)	16 (2.2)		194 (25.3)	10 (3.1)		15 (4.6)		6 (1.5)		
	11q23	231 (21.3)	173 (23.7)		236 (21.6)	168 (23.3)		145 (18.9)	86 (27)		91 (27.9)		82 (20.3)		
	Normal	181 (16.7)	228 (31.3)		246 (22.5)	163 (22.6)		120 (15.7)	61 (19.2)		126 (38.7)		102 (25.3)		
	Other	200 (18.5)	307 (42.1)		203 (18.6)	304 (42.2)		109 (14.2)	91 (28.6)		94 (28.8)		213 (52.9)		
FLT3 status	Wild type	1010 (90.8)	573 (76.8)	**<0.0001**	953 (85.7)	630 (84.5)	0.4654	712 (91)	298 (90.3)		241 (73)		332 (79.8)		**<0.0001**
	ITD/Mutation	102 (9.2)	173 (23.2)		159 (14.3)	116 (15.5)		70 (9)	32 (9.7)		89 (27)		84 (20.2)		
WBC group	<50	707 (63.5)	474 (63.9)	0.8803	669 (60.1)	512 (69)	**<0.0001**	479 (61.1)	228 (69.1)		190 (57.6)		284 (68.9)		**0.0012**
	≥50	407 (36.5)	268 (36.1)		445 (39.9)	230 (31)		305 (38.9)	102 (30.9)		140 (42.4)		128 (31.1)		
Induction I response	CR	450 (83.5)	257 (70.8)	**<0.0001**	437 (81.1)	270 (74.4)	**0.0169**	328 (85.6)	122 (78.2)		109 (69.9)		148 (71.5)		**<0.0001**
	Not CR	89 (16.5)	106 (29.2)		102 (18.9)	93 (25.6)		55 (14.4)	34 (21.8)		47 (30.1)		59 (28.5)		
Induction II response	CR	390 (94)	228 (82.3)	**<0.0001**	374 (90.1)	244 (88.1)	0.4538	275 (94.5)	115 (92.7)		99 (79.8)		129 (84.3)		**<0.0001**
	Not CR	25 (6)	49 (17.7)		41 (9.9)	33 (11.9)		16 (5.5)	9 (7.3)		25 (20.2)		24 (15.7)		
Induction I MRD	Negative	740 (82.2)	345 (56.8)	**<0.0001**	712 (78.5)	373 (62.2)	**<0.0001**	532 (84)	208 (77.9)		180 (65.7)		165 (49.5)		**<0.0001**
	Positive	160 (17.8)	262 (43.2)		195 (21.5)	227 (37.8)		101 (16)	59 (22.1)		94 (34.3)		168 (50.5)		
Induction II MRD	Negative	728 (92.4)	387 (77.4)	**<0.0001**	695 (89.3)	420 (82.4)	**0.0004**	516 (93)	212 (91)		179 (80.3)		208 (75.1)		**<0.0001**
	Positive	60 (7.6)	113 (22.6)		83 (10.7)	90 (17.6)		39 (7)	21 (9)		44 (19.7)		69 (24.9)		
5-year EFS		54.61 (1.51)	31.24 (1.73)	**<0.0001**	50.68 (1.52)	37.15 (1.8)	**<0.0001**	57.76 (1.78)	47.04 (2.79)		33.73(2.65)		29.27 (2.29)		**<0.0001**
5-year OS		72.07 (1.38)	48.57 (1.91)	**<0.0001**	68.72 (1.43)	53.73 (1.89)	**<0.0001**	75.42 (1.58)	64.12 (2.71)		52.59 (2.86)		45.39 (2.54)		**<0.0001**
Adult AML Validation Cohort: Total *N* = 1669															
Gender	Female	456 (45.6)	330(49.4)	0.1359	451 (45.1)	335 (50.1)	**0.0426**	326 (45.3)	126 (49)		121 (47.1)		209 (50.9)		0.3255
	Male	545 (54.4)	338(50.6)		550 (54.9)	333 (49.9)		393 (54.7)	131 (51)		136 (52.9)		202 (49.1)		
Age group	<65	799 (79.8)	516(77.2)	0.2253	793 (79.2)	522 (78.1)	0.6227	577 (80.3)	204 (79.4)		198 (77)		318 (77.4)		0.5946
	≥65	202 (20.2)	152(22.8)		208 (20.8)	146 (21.9)		142 (19.7)	53 (20.6)		59 (23)		93 (22.6)		
Risk group	Favorable	300 (30.4)	35(5.3)	**<0.0001**	256 (25.8)	79 (12.1)	**<0.0001**	241 (33.8)	56 (22.4)		12 (4.7)		23 (5.7)		**<0.0001**
	Intermediate	469 (47.5)	392(59.6)		475 (47.8)	386 (59.2)		324 (45.5)	134 (53.6)		140 (54.7)		252 (62.7)		
	Adverse	218 (22.1)	231(35.1)		262 (26.4)	187 (28.7)		147 (20.6)	60 (24)		104 (40.6)		127 (31.6)		
Cytogenetic group	t(8;21)	63 (11.7)	0(0)	**<0.0001**	43 (8)	20 (5.6)	**<0.0001**	43 (10.6)	20 (15.2)		0 (0)		0(0)		**<0.0001**
	inv(16)	73 (13.6)	3(0.8)		72 (13.4)	4 (1.1)		70 (17.3)	3 (2.3)		2 (1.5)		1 (0.4)		
	11q23	29 (5.4)	19(5.3)		29 (5.4)	19 (5.3)		22 (5.4)	7 (5.3)		7 (5.2)		12 (5.3)		
	Normal	210 (39.1)	181(50.1)		220 (40.8)	171 (47.6)		158 (39)	52 (39.4)		62 (46.3)		119 (52.4)		
	Other	162 (30.2)	158(43.8)		175 (32.5)	145 (40.4)		112 (27.7)	50 (37.9)		63 (47)		95 (41.9)		
FLT3 status	Wild type	782 (79.6)	336(51.1)	**<0.0001**	725 (73.2)	393 (60.4)	**<0.0001**	557 (78.5)	202 (81.5)		145 (56.9)		19 1(47.4)		**<0.0001**
	ITD/Mutation	201 (20.4)	322(48.9)		265 (26.8)	258 (39.6)		153 (21.5)	46 (18.5)		110 (43.1)		212 (52.6)		
WBC group	<50	513 (70.1)	323(66.6)	0.2162	502 (68.8)	334 (68.6)	0.9492	354 (68.9)	143 (74.1)		132 (69.1)		191 (65)		0.2112
	≥50	219 (29.9)	162(33.4)		228 (31.2)	153 (31.4)		160 (31.1)	50 (25.9)		59 (30.9)		103 (35)		
5-year EFS		31.01 (1.68)	13.01(1.51)	**<0.0001**	26.53 (1.61)	19.79 (1.77)	**0.0012**	32.06 (2.01)	28.98 (3.24)		11.39 (2.29)		13.94 (1.99)		**<0.0001**
5-year OS		40.26 (1.64)	16.73(1.54)	**<0.0001**	35.13 (1.6)	24.46 (1.76)	**<0.0001**	43.15 (1.95)	33.16 (3.09)		13.17 (2.26)		18.98 (2.07)		**<0.0001**

Race was not available in the adult cohorts; Discovery cohort AML02 is represented in Supplementary Table 3 and not included in the Pediatric combined dataset; GSE71014 dataset of CN patients from Taiwan was not added to the combined adult datasets analysis.

*EFS* event free survival, *OS* overall Survival, *CR* complete remission.

**Fig. 3:**
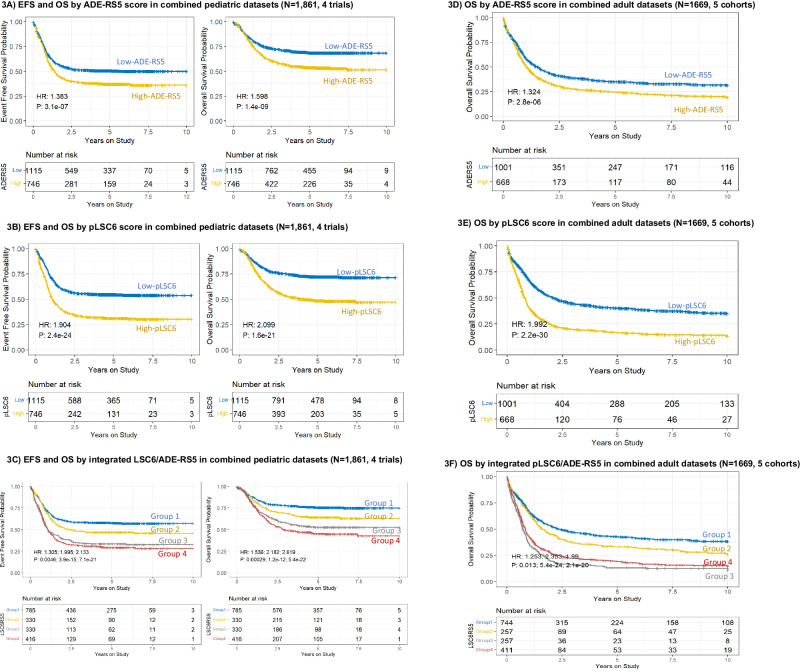
ADE-RS5, pLSC6 and integrated score groups predict EFS and OS in large pediatric and adult AML validation cohorts. Association of ADE-RS5 (**A**), pLSC6 (**B**), and the integrated LSC6/ADE-RS5 four score groups (**C**) with EFS and OS in the combined pediatric AML validation cohorts from multiple multi-site clinical trials (*N* = 1861, 4 trials). Association of ADE-RS5 (**D**), pLSC6 (**E**), and the integrated LSC6/ADE-RS5 four score groups (**F**) with OS in the combined adult AML validation cohorts from multiple multi-site clinical trials (*N* = 1669 patients, 5 cohorts).

**Fig. 4:**
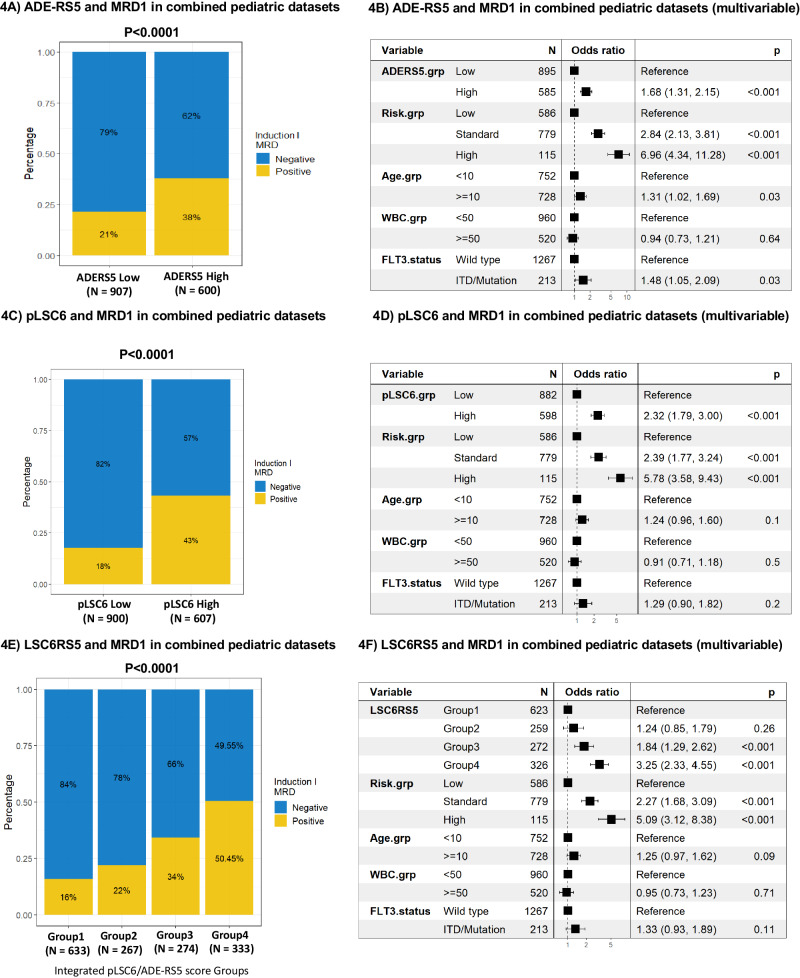
ADE-RS5, pLSC6 and integrated score groups predict MRD after induction 1 in large pediatric AML validation cohorts. Association of ADE-RS5 (**A**), pLSC6 (**C**), and the integrated LSC6/ADE-RS5 four score groups (**E**) with MRD1 in 1507 pediatric AML patients (COG-cohort1, COG-cohort2, and AML08 datasets). Forest plots showing results of multivariable cox regression analysis of association of ADE-RS5 (**B**), pLSC6 (**D**), and the integrated score groups (**F**) and MRD1 after adjusting for risk group assignment, diagnostic WBC count, FLT3 status and age. * MRD1 data was not available from the pediatric GSE17855 dataset.

In the combined pediatric cohort (*n* = 1861), EFS and OS showed significant and consistent association for ADE-RS5 (EFS: HR = 1.38 and OS: HR = 1.6, both *p* < 0.001; Fig. 3A), pLSC6 (EFS: HR = 1.9, and OS: HR = 2.1, both *p* < 0.001; Fig. 3B). For integrated pLSC6/ADE-RS5 group analysis with Group 1 being reference both EFS and OS showed inferior outcome in other groups (EFS: HR = 1.31, *p* = 0.005 (Group 2 vs Group 1), HR = 1.99, *p* < 0.001 (Group 3 vs Group 1) and HR = 2.13, *p* < 0.001 (Group 4 vs Group 1); OS: HR = 1.54 (Group 2 vs. Group 1), HR = 2.18 (Group 3 vs. Group 1), and HR = 2.62 (Group 4 vs Group1) all *p* < 0.001; Fig. 3C). Endpoint associations are also summarized in Table 1. In multivariable analysis after adjusting for cytogenetics risk group, age and WBC count in the combined pediatric AML patient population, ADE-RS5 was not significantly associated with ESF (*p* = 0.3) and OS (p = 0.06) however pLSC6 showed consistent significant association with EFS (p < 0.001) and OS (*p* < 0.001) (Supplementary Fig. 3A, B). For integrated pLSC6/ADE-RS5 score, groups 3 and 4 consistently showed significantly association with poor EFS and OS after adjusting for age, risk group and WBC as compared to group 1 (Supplementary Fig. 3C). Given that MRD after induction I holds prognostic value in driving the clinical decisions, we analyzed pLSC6 and ADE-RS5 scores with MRD1 data which was available in 3 of the 4 cohorts. ADE-RS5, pLSC6 and integrated score groups showed consistent and significant association with MRD1 (MRD1 positivity: ADER-RS5, high vs. low: 38% vs. 21%; pLSC6, high vs. low, 43% vs. 18% and for integrated score groups, 16% of group 1, 22% of group 2, 34% of group 3 and 50% of group 4 patients were MRD1 positive, all *p* < 0.0001, Figs. 4A, C, E, respectively). Individual and integrated score groups remained significant predictors of MRD1 in multivariable logistic regression models after adjusting for age, risk group, WBC and FLT3 status (ADE-RS5; OR = 1.68, *p* < 0.001, pLSC6; OR = 2.32, *p* < 0.001, for integrated score groups; using group 1 as reference, group 2: OR = 1.24, *p* = 0.26, group 3: OR = 1.84, *p* < 0.001, group 4: OR = 3.25, *p* < 0.001 Fig. 4B, D, F, respectively).

Within adult AML, we investigated 1669 patients from 5 different cohorts (OS data was available in all cohorts, but EFS was available on only 3 cohorts). In the combined cohort analysis of patients from 5 different trials, OS showed significant and consistent association with ADE-RS5 score (OS: HR = 1.32, *p* < 0.001; Fig. 3D), pLSC6 (OS: HR = 1.99, *p* < 0.001; Fig. 3E), and integrated pLSC6-ADE-RS5 score groups (Group 2 vs.1: OS: HR = 1.25, *p* = 0.013 Group 3 vs. 1: HR = 2.35, *p* < 0.001 and Group 4 vs. 1: HR = 1.99, *p* < 0.001 Fig. 3F). Multivariable analysis after adjusting for risk group, age, and *FLT3*-ITD mutation, ADE-RS5, pLSC6 and integrated pLSC6 and ADE-RS5 score groups remained significant predictors of OS (Supplementary Fig. 3D–F). For cohorts with EFS data available (*n* = 1306), consistent significant associations were observed for the ADE-RS5 (HR = 1.23, *p* = 0.001), pLSC6 (HR = 1.86, *p* < 0.001) and integrated score groups (Group 3 vs. 1 and Group 4 vs. Group 1, *p* < 0.001) (Supplementary Fig. 4A, C, E). In multivariable analysis adjusting for age, risk group and *FLT3*-ITD mutation, pLSC6 and integrated score groups remained significant predictors of EFS (Supplementary Fig. 4B, D, F).

Age stratified analysis for adults less than 65 years old and elderly patients who are ≥65 years old showed pLSC6 (pLSC6 low vs. high, <65 yrs, HR = 2.06, *P* < 0.00001; ≥65 yrs, HR = 2.02, *P* < 0.00001, Supplementary Fig. 5A, C), and ADE-RS5 (low ADE-RS5 vs. high, <65 y, HR = 1.37, *P* < 0.00001, and ≥65 yrs, HR = 1.21, *p* = 0.093, Supplementary Fig. 5E, F) to be associated with OS. The integrated scores remained a significant predictor of OS in the two age groups (Supplementary Fig. 5I, K). In the multivariable analysis adjusting for risk group assignment and *FLT3*-ITD mutation, pLSC6 and the integrated scores remained as significant independent predictor of OS in both age groups (Supplementary Fig. 5). Given cytogenetically normal (CN) subgroup of AML patients constitute significant proportion of patients and experience highly heterogenous response, we evaluated ADE-RS5, pLCS6 and integrated scores within these subgroups in all the 9 cohorts as well as in an additional cohort of CN patients from *GSE71014* dataset. Consistent with the results from the whole cohort within CN-AML with high-pLSC6/high ADE-RS scores experienced significantly lower EFS and OS compared to low-pLSC6/low ADE-RS score group in pediatric and adult cohorts (Supplementary Fig. 6A, B). Multivariable analysis adjusting for age, WBC count at diagnosis and FLT3-status, pLSC6, ADE-RS5, and integrated score groups remained significant independent predictors of outcomes in pediatric and adult CN patients (Supplementary Fig. 6A, B).

Additionally, hematopoietic stem cell transplant (HSCT) can have a significant impact on outcome and we previously showed that patients with high pLSC6 score do not show benefit from HSCT in AML02 cohort^7^. Though HSCT information was not available in all cohorts we evaluated HSCT as a time-dependent variable for pLSC6, ADE-RS5 and the integrated score in 4 cohorts with availability of data. As shown in Supplementary Fig. 7, the score groups remained significant predictor of EFS and OS.

**Fig. 5:**
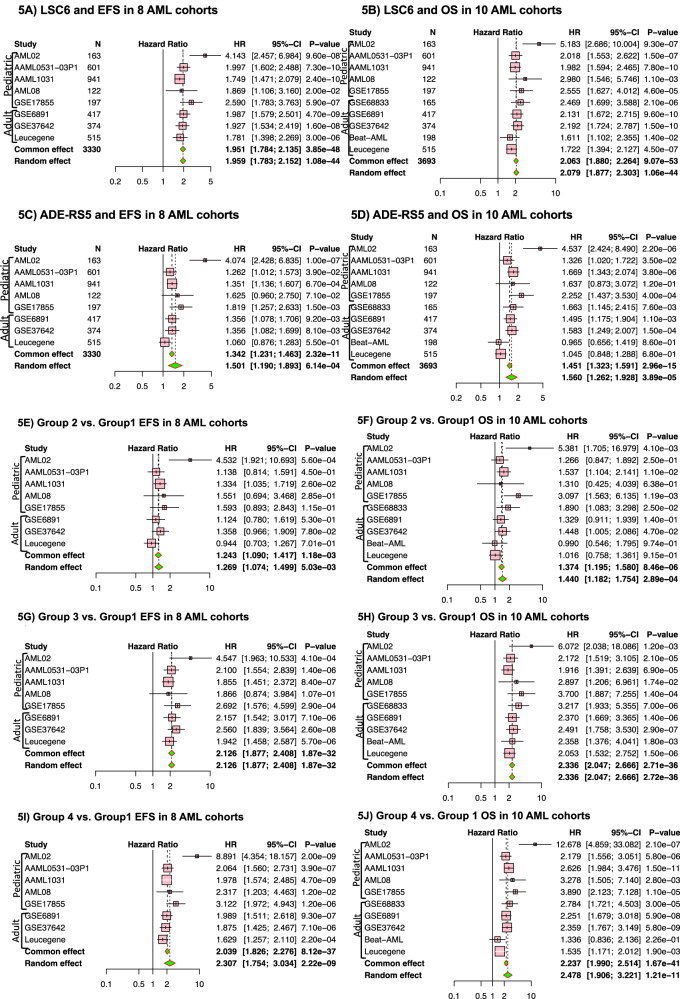
Metanalysis forest plots for ADE-RS5, pLSC6 and integrated score groups in 10 AML cohorts. Meta-analysis of EFS in 8 pediatric and adult AML datasets and OS in 10 pediatric and adult AML datasets by pLSC6 (**A**, **B**), ADE-RS5 (**C**, **D**), and integrated LSC6/ADERS scores group 1 vs. 2 (**E**, **F**), group 1 vs. group 3(**G**, **H**), group 1 vs. group 4 (**I**, **J**).

In addition to the analysis performed in the combined cohorts for pediatric and adult AML, each cohort was evaluated individually. Figure 5 shows a summary of results for association of both pLSC6 and ADE-RS5 scores in individual cohorts for EFS (5 pediatric cohorts) and 3 adult AML cohorts, (*N* = 3330) and OS (5 pediatric and 5 adult AML cohorts, total *N* = 3693). Consistent with the results from the discovery cohort pLSC6 was significantly associated with EFS (Fig. 5A) and OS (Fig. 5B) in all individual cohorts tested with common effect of HR = 1.95, 95%CI = 1.78–2.14, *p* < 0.00001 for association with EFS, and HR = 2.06, 95%CI = 1.88–2.26, *P* < 0.00001 for association with OS. ADE-RS5 was significantly associated with EFS in all cohorts (*p* < 0.01) except for AML08 (*p* = 0.07) and the Leucegene (*p* = 0.55) cohort, and with OS in all cohorts (*p* < 0.01) except for AML08 (*p* = 0.12), Beat AML (*p* = 0.8) and the Leucegene (*p* = 0.68) cohort, with common effect of HR = 1.34, 95%CI = 1.23–1.46, *p* < 0.00001 for association with EFS, and HR = 1.45, 95%CI = 1.32–1.59, *p* < 0.00001 for association with OS (Fig. 5C, D). Figure 5E–J shows the results for integrated LSC6-ADE-RS5 score (Groups 2–4 vs. Group 1) again showing Group 4 with worst outcome as compared to Group 1.

## Discussion

Cytarabine, daunorubicin and etoposide (ADE) are commonly used for induction of remission and intensification of pediatric AML. A combination of cytarabine and anthracyclines is the mainstay of treatment in adults. However, development of chemotherapeutic resistance is a major cause of AML treatment failure^3,5^. In recent years, significant effort has been devoted on transcriptomics based prognostic factors including leukemic stemness score (LSC17) reported in 2016^6^ in an adult AML. Our group had previously leveraged the leukemia stemness genes identified by Ng et al. ^6^ and using outcome data from pediatric AML developed a pediatric leukemic stemness score that composed of 6 genes^7^. In addition to leukemic stemness that defines disease aggressiveness, development of drug resistance is an inherent clinical challenge. In this study, we used similar strategy to define a chemotherapeutic resistance score focused on key genes of pharmacological relevance (pharmacokinetics/ pharmacodynamics) to ADE. After running LASSO regression key genes of pharmacological relevance to ADE, we defined an ADE-RS score that was computed for each patient based on the expression level of five genes multiplied by their regression coefficients. These five genes included (i) deoxycytidylate deaminase (*DCTD*), a deaminase involved ara-CMP to ara-UMP conversion; (ii) ATP Binding Cassette Subfamily C Member 1 (*ABCC1*), an efflux transporter implicated in daunorubicin and etoposide efflux; (iii) Myeloperoxidase (*MPO*), involved in etoposide-catechol to quinone conversion^9^, MPO is also a myeloid cell specific marker^10^; (iv) Topoisomerase II alpha (*TOP2A*), daunorubicin and etoposide target^11^; and (v) Carbonyl Reductase 1 (*CBR1*), involved in reduction of daunorubicin to daunorubicinol^12,13^. Drug metabolism is a very complicated process with involvement of influx, efflux transporters, activating and inactivating enzymes and the dynamic interaction between these making it very challenging to simultaneously study all of these. Thus, alternative approaches as are done here provide some insight into drug responsiveness governed by pharmacological genes. To the best of our knowledge this is one of the first studies to apply this approach to establish drug resistance score that holds prognostic value and is predictive of survival outcomes.

Further a previously established pLSC6 and newly developed ADE-RS5 score were evaluated as a prognostic factor in 9 independent pediatric and adult AML cohorts totaling more than 3000 patients. pLSC6 score was validated in each cohort and within cytogenetically normal group as well as within patients less than and more than 65 yrs old. This is in contrast to recent observation where LSC17 was not associated with EFS and OS in patients ≥60 yrs age^14^.

Furthermore, the ADE-RS5 score predicted outcome within low and high pLSC6 groups indicating it offers additional prognostic value beyond that captured by the pLSC6 score alone. Thus, a four-group classifier system (Group 1 to Group 4) was developed for patients. Integrated stemness and drug-resistance score groups predicted outcome in both pediatric and adult AML patients as well as within different cytogenetic subgroups as well as within CN-AML. Group 1 representing patients with low-LSC6 and low-ADE-RS5 group had the most favorable outcome and group 4 with both high scores had the poorest outcomes. In addition, both pLSC6, ADE-RS5, and the integrated score groups, were significant and independent predictors of poor outcomes after adjusting for risk group assignment, age, *FLT3*-ITD mutation and WBC count at diagnosis. ADE-RS5 is not validated in BEAT AML and Leucegene cohorts and we believe this may be due to older age of the patient’s, different frequency of cytogenetic risk categories, treatment regimens without etoposide, and potential effect of transplant. Gene expression levels of all genes that are part of LSC17 was not available in all cohorts due to the type of array used, however we evaluated LSC17 groups as previously described and stayed significant predictor of OS. Combination of ADE-RS5 and LSC17 showed added value of ADE-RS5 in predicting survival (Supplementary Fig. 8). Despite this being one of the few studies with large patients’ samples across multiple cohorts there are some limitations such as non-uniform treatment protocols across the cohorts, continued updates on the AML classification resulting in changes in the initial risk group classification in older trials, variability in the post-induction treatment protocols across trials and centers, lack of availability of EFS data and time to transplant in some adult cohorts, lack of mechanistic studies supporting functional relevance of some of the genes that are part of the score.

In conclusion, this report highlights the significant prognostic value of multi-gene transcriptomics-based scores, that includes the assessment of disease aggressiveness through pLSC6 score and drug resistance via ADE-RS5 score. Our analysis reveals that the pLSC6 stemness score is a significant predictor of outcome and associates with high-risk group features, the ADE-RS5 drug resistance score adds further value, reflecting the clinical utility of simultaneous testing of both to optimize treatment strategies. One notable aspect of this study is the evaluation of nine entirely independent clinical cohorts, including both pediatric and adult AML patients from various countries. Evaluation of only 6 genes highlights the simplicity of clinical utility of pLSC6. Future clinical translation of these results, can be accelerated by use of a simple method for quantification of 11 genes such as that based on RT-PCR or use of nano string based assay, we have previously shown consistency for pLSC6 score across three platforms U133A, RNAseq and RT-PCR^7^. Future work is focused on developing a web-based tool that will allow for other investigators to utilize our signatures to predict treatment outcomes and refining patient classification.

## Methods

### Patient cohorts- AML02 discovery cohort

For this study, we included 163 patients treated on the multicenter AML02 clinical trial (ClinicalTrials.gov Identifier: NCT00136084). Patients with acute promyelocytic leukemia or Down’s syndrome were excluded, patient characteristics, risk group assignment and definition of clinical endpoints including minimal residual disease after induction I course of the treatment (MRD1), event-free survival (EFS) and overall survival (OS) have been previously described^15^. Gene expression profiling of leukemic blasts obtained at diagnosis in the AML02 discovery cohort was performed using GeneChip® Human Genome U133A [Affymetrix, Santa Clara, CA] as described previously^16^. The MAS 5.0 algorithm was used to obtain normalized gene expression signals. Expression data for 67 genes of relevance to ADE pharmacology (listed in Supplementary Table S1) was extracted and log2 transformed before the analysis.

### Validation cohorts

AML patient cohorts with both gene expression data from diagnostic specimen and clinical outcome data available were included in the validation studies. Patients diagnosed with myelodysplastic syndrome (MDS), myelodysplastic syndrome refractory anemia with excess blasts (MDS-RAEB), Down’s syndrome-related AML and acute promyelocytic leukemia (APL; FAB-M3), data from specimens not from diagnosis or those missing survival data were excluded from the study. The validation cohorts are summarized below and listed in Fig. 1 (additional details are provided in the Supplementary Material). All the cohorts were evaluated for association between transcriptomic scores and clinical outcome endpoints individually as well as in a combined into pediatric and adult AML datasets. Use of data and/or specimens were approved by the respective protocol or institutional Institutional Review Boards, and informed consent was obtained from parents/guardians or patients and assents from the patients, as appropriate, in accordance with the approved clinical trial protocols and in accordance with Helsinki declaration. Study was approved by University of Florida Institutional Review Board.

#### Pediatric AML-children’s oncology group (COG) AAML0531 and AAML03P1

This dataset included 601 pediatric AML patients treated under the COG AAML0531^17^ (NCT00372593; *N* = 531) and AAML03P1 (NCT0070174; *N* = 70)^18^ trials. Details on the clinical trial and outcome have been previously published^17,19^. The RNAseq and clinical outcome data was provided by COG or downloaded through TARGET-AML project dataset (https://ocg.cancer.gov/programs/target/projects/acute-myeloid-leukemia).

#### Pediatric AML -children’s oncology group (COG) AAML1031

This dataset included 941 pediatric AML patients treated under the COG-AAML1031 (NCT01371981). RNAseq and clinical outcome data provided by COG or obtained from TARGET-AML project (https://ocg.cancer.gov/programs/target/projects/acute-myeloid-leukemia). Details on the clinical trial and outcome have been previously published^20^.

#### Pediatric AML-AML08 cohort

This dataset included 122 pediatric AML patients treated under the multi-center AML08 clinical trial (NCT00703820) and were included in this evaluation^21^. RNA samples from diagnosis were available from 122 patients and gene expression data on 11 genes of interest was generated using Taqman based assay as detailed in Supplementary Material. Details on the clinical trial and outcome have been previously published^21^.

#### Pediatric AML-GSE17855 cohort

For this cohort, data from 197 pediatric AML patients (following exclusion criteria listed above) were included in this study. Patients received treatment on 8 different trials. Expression data generated using U133 plus array was downloaded from Gene Expression Omnibus (GSE) database (GSE17855).

#### Adult AML-GSE68833- the cancer genome atlas (TCGA) cohort

This dataset included 165 adult AML patients with publicly available clinical and gene expression data. U133-Plus microarray gene expression data was downloaded for this group of patients from Gene Expression Omnibus database (GSE68833). RNA-Seq gene expression data for 153 patients was also available for this cohort.

#### Adult AML-GSE37642

This dataset included 374 adult AML patients treated in the German AMLCG-1999 trial^22^ with publicly available gene-expression data generated using U133A array^23^.

#### Adult AML-GSE6891

This dataset included 417 adult AML patients treated according to sequential Dutch-Belgian Hemato-Oncology Cooperative Group and the Swiss Group for Clinical Cancer Research multiple HOVON trails with publicly available gene expression data generated using U133 plus array.

#### Adult AML-BeatAML

Clinical data was downloaded from http://www.vizome.org/aml/ and merged with clinical data downloaded from C-bioportal-OHSU^24^. After applying exclusion criteria indicated above, 198 patients were included in the current study.

#### Adult AML-Leucegene AML cohort

This dataset included 515 adult patients with newly diagnosed AML who were treated with intensive induction chemotherapy (7 + 3 based regimens) in Quebec (Canada) between 2001 and 2019. Diagnostic bone marrow or peripheral blood samples were collected and stored by the Quebec leukemia cell bank (bclq.org). Gene expression data was generated with whole transcriptome sequencing using an Illumina HiSeq 2000 sequencing system as part of the Leucegene project (leucegene.ca) and clinical data was collected and validated by the Quebec leukemia cell bank (details in supplementary material).

#### Adult AML-GSE71014- Cytogenetically normal AML dataset

Cytogenetically normal AML (CN-AML) patients (*n* = 104) treated at the National Taiwan University Hospital (NTUH)^25^ with gene-expression and clinical data available (HumanHT-12 V4.0 expression bead chip).

All the gene expression data was log2 transformed before analysis. RNA-Seq data was normalized as Reads per kilo base of transcript per million mapped reads (RPKM) or transcripts per million (TPM). We used log2 (RPKM + 1) or log2 (TPM + 1) values for subsequent statistical analysis. Supplementary Table 2 provides a list of probe/assay IDs for the 11 genes that constitute pLSC6 and ADE-RS5 score.

### Clinical Outcome endpoint definitions

Minimal residual disease after induction I course (MRD1) of treatment was defined as one or more leukemic per 1000 mononuclear cells (≥0.1%). Event-free survival (EFS) was defined in the AML02 discovery cohort as the time from study enrollment to induction failure, relapse, second malignancy, refusal of therapy, removal from therapy because of unacceptable toxicity, or death, with patients who had not experienced any of these events censored at last follow-up. The definition of EFS among other clinical trials is described in the respective clinical trial outcome reports cited above or in supplemental information. Overall survival (OS) was defined as the time from study enrollment to death, with living patients censored at last follow-up.

### Development of ADE-RS score

We utilized a least absolute shrinkage and selection operator (LASSO) Cox regression model, as implemented in glmnet package of the R3.6.0 statistical software (www.r-project.org), to the gene expression levels (67 genes of pharmacological relevance to ADE) and the EFS data of patients from the AML02 discovery cohort. To evaluate the variability and reproducibility of the LASSO Cox regression model estimates, we repeated the LASSO Cox regression fitting process for each of 1,000 leave-10%-out cross-validation evaluations. Genes with non-zero coefficient estimates in at least 950 of these 1000 evaluations were retained. The final model coefficient was obtained by averaging the coefficient estimates obtained for the set of cross-validation evaluations. We further utilized a recursive partitioning survival model, as implemented in the rpart package, to dichotomize ADE-resistance scores into “low” and “high” score groups (60% as low and 40% as high).

### Integrated pLSC6/ADE-RS5 score groups

pLSC6 score was generated based on the expression level of six genes: *DNMT3B, GPR56, CD34, SPINK2, SOCS2, FAM30A* multiplied by their regression coefficients as defined previously^7^. Patients were classified as low or high pLSC6 groups as defined previously. Based on combination of the pLSC6 and ADE-RS5 score group designation, patients were further grouped as described in the results section. Association between pLSC6, ADE-RS5, and integrated score groups with clinical outcome endpoints was analyzed on the individual cohort level of pediatric AML datasets that included COG-cohort 1 (*N* = 601), COG-cohort 2 (*N* = 941), AML08 (*N* = 122) and GSE17855 (*N* = 197), and in the combined pediatric totaling 1861 patients. Similarly, we analyzed validation adult AML datasets individually in GSE68833-TCGA (*N* = 165), GSE37642 (*N* = 374), GSE6891 (*N* = 417), Beat-AML (*N* = 198), Leucegene (*N* = 515) cohorts as well as in the combined cohort totaling 1669 adult AML patients.

### Statistical analysis

Survival analyses were performed using survival and survminer packages in R3.6.0. EFS and OS probabilities were estimated using the Kaplan-Meier method and Cox proportional hazard models was used to compare the survival curves of patients within ADE-RS5, pLSC6 and integrated pLSC6/ADE-RS5 score groups (Groups 1–4) as well as the association between each individual prognostic factor and survival outcomes. Multivariable Cox proportional hazards model was used to evaluate the independent prognostic effect of the study covariables. Wilcoxon rank-sum or Kruskal-Wallis tests was used for continuous variable comparisons between/among patient subgroups. Chi-square or fisher exact tests were used for testing association between categorical variables. For the meta-analysis, HRs and their 95% CIs were from Cox proportional hazard model with or without adjustment of known factors in individual cohorts. The overall HRs were estimated using meta-analysis (meta_6.1-0) with fixed effect model. The overall HRs were also provided with random effects allowing for heterogeneity among cohorts. Heterogeneity could be evaluated by I^2^. All analyses were conducted in R Statistical software version 3.6.0 (R Foundation for Statistical Computing, Vienna, Austria) R-4.2.1, and a two-tailed *P* value less than 0.05 was deemed statistically significant. The R script codes are available at GitHub (https://github.com/Abdelrahman-Elsayed/kit-nfold-cv-glmnet/blob/master/kit-nfold-cv-glmnet-v0.R). A stepwise model development flow chart is provided in Supplementary Material.

### Supplementary information

Supplemental Material

## Data Availability

The data used in the validation cohorts is available at the sources cited with each cohort.
