# Supplementary material for: Integrated drug resistance and leukemic stemness gene-expression scores predict outcomes in large cohort of over 3500 AML patients from 10 trials
Source: NPJ Precis Oncol. 2024 Aug 1;8:168. doi: 10.1038/s41698-024-00643-5 (PMC11294346; doi:10.1038/s41698-024-00643-5)
Supplement: Supplementary file 1 — Supplemental Material [file 41698_2024_643_MOESM1_ESM.pdf]

Supplementary Materials:

Validation Cohorts: AML patient cohorts with both gene expression data from diagnostic specimen and clinical outcome data available were included in the validation studies. Few exclusion criteria included patients diagnosed with myelodysplastic syndrome (MDS), myelodysplastic syndrome refractory anemia with excess blasts (MDS-RAEB), Down's syndrome-related AML and acute promyelocytic leukemia (APL; FAB-M3) were excluded from the study. Patients with missing survival data and patients who died or left the clinical trial before receiving any treatment were also excluded from the study. The validation cohorts are listed in Figure 1 and the details are provided in the Supplementary Material.

1. **Children's Oncology Group (COG) cohort 1:** This dataset included 601 pediatric AML patients treated under the Children's Oncology Group (COG) AAML0531**(1)** (NCT00372593; N=531) and AAML03P1 (NCT0070174; N=70) **(2) trial**s. Details on the clinical trial and outcome have been previously published**(1, 3).**The RNAseq and clinical outcome data was provided by COG or downloaded through TARGET-AML project dataset (https://ocg.cancer.gov/programs/target/projects/acute-myeloid-leukemia).
COG cohort 1- RNAseq processing: RNA-Seq data in the COG validation cohort was processed and normalized using two different approaches. Expression data for 421 patients provided by our collaborators at Fred Hutchinson Cancer Center was normalized as Transcripts per Million reads (TPM). On the other hand, publicly available expression data for 180 patients on TARGET-AML database was normalized as Reads per kilobase of transcript per million mapped reads (RPKM). To account for such disparate metrics, after computing the ADE-RS and pLSC6 scores; we performed Z-transformation for the scores in each dataset separately before combining scores from the two datasets. Z-transformation is a standardization or auto-scaling method to compare scores from disparate distributions.(4) After applying Z-transformation, each score will have a standardized distribution (mean = 0 and standard deviation = 1), allowing for comparison of scores with different distributions.
2. **Children's Oncology Group (COG) cohort 2:** This dataset included 941 pediatric AML patients treated under the COG-AAML1031 (**NCT01371981)** trial with patients in this trial randomized to receive standard treatment with or without bortezomib. RNAseq and clinical outcome data either provided by COG or obtained from TARGET-AML project (https://ocg.cancer.gov/programs/target/projects/acute-myeloid-leukemia). Details on the clinical trial and outcome have been previously published(5).
3. **AML08 Cohort:** This dataset included 122 pediatric AML patients treated under the multi-center AML08 clinical trial (NCT00703820), were included in this evaluation. In AML08 trial patients were randomized to receive either clofarabine and cytarabine on days 1–5 (clo/ara-C arm) or high-dose cytarabine with daunorubicin and etoposide in the first induction course of the treatment(6). RNA samples from diagnosis were available from 122 patients and gene expression data on 11 genes of interest was generated using Taqman based assay. Details on the clinical trial and outcome have been previously published(6). Expression of the 11 genes that are part of pLSC6 and ADE-RS score equations were determined using TaqMan® Low-Density Array (TLDA) cards (Applied Biosystems, USA) as per instructions. Diagnostic RNA sample for each patient in AML08 cohort was reverse transcribed into single-stranded DNA followed by complementary DNA (cDNA) synthesis using High-Capacity cDNA Reverse Transcription Kit (Applied Biosystems, USA). Gene expression of the target genes was normalized (to geometric mean of housekeeping genes *GAPDH* and *ACTB*) using dCT method. ddCT method was used to determine relative quantification of the 11 genes.
4. **GSE17855 cohort:** For this cohort data from 197 patients (following exclusion criteria listed above) were included. Patients received treatment on 8 different trials. Expression data generated using U133 plus array was downloaded from GEO database (GSE17855) and clinical data was provided by Dr. Zwaan.
5. **GSE68833- The Cancer Genome Atlas (TCGA) cohort:** This dataset included 165 adult AML patients with publicly available clinical and gene expression data. U133-Plus microarray gene expression data was downloaded for this group of patients from Gene Expression Omnibus database (GSE68833). RNA-Seq gene expression data for 153 of the same165 patients in the TCGA cohort was downloaded from TCGA-Firebrowse database (http://firebrowse.org/). Patient demographics such as gender, race, and age at diagnosis in addition to risk group assignment, FAB classification, WBC count at doagnosis and mutation status of genes commonly mutated in AML such as *FLT3*, *NPM1*, *DNMT3A* and *TP53 was downloaded* (https://gdc.cancer.gov/about-data/publications/laml_2012 - supplemental table 1)(7).
6. **GSE37642:** This dataset included 374 adult AML patients with publicly available gene expression data (U133-A microarray gene expression data was downloaded from GEO:GSE37642). Patients were treated in the German AMLCG-1999 trial (8, 9).
7. **GSE6891:** This dataset included 417 adult AML patients with publicly available gene expression data (U133 plus 2 array GEO: GSE6891). Patients were treated according to sequential Dutch-Belgian Hemato-Oncology Cooperative Group and the Swiss Group for Clinical Cancer Research on multiple HOVON trails.
8. **GSE71014** dataset of cytogenetically normal patients (n=104). Cytogenetically normal (CN) *de novo* AML patients treated from 1995 – 2011 at the National Taiwan University Hospital (NTUH)(10). Gene expression and overall survival data was downloaded from GEO database (HumanHT-12 V4.0 expression beadchip GSE71014). Illumina was used for gene expression profiling and normalized data was log2 transformed before analysis. If the same gene is represented by multiple probes on the beadchip, we used the probe with the highest average expression among patients in the dataset.
9. **Leucegene AML prognostic cohort:** This dataset included 515 adult patients with newly diagnosed AML who have been treated with intensive induction chemotherapy (7+3 based regimens) across 10 different centers in Quebec (Canada) between 2001 and 2019. Diagnostic bone marrow or peripheral blood samples were collected and stored by the Quebec leukemia cell bank (bclq.org). Mononuclear cells were prepared from samples using Ficoll and cryopreserved in TRIzol or in liquid nitrogen (DMSO 10%). For sequencing preparation, cells were thawed and RNA was extracted using TRIzol reagent according

to the manufacturer's instruction (Invitrogen / Life Technologies) with additional purification on RNeasy mini columns (Qiagen). Gene expression data was generated with whole transcriptome sequencing using an Illumina HiSeq 2000 sequencing system as part of the Leucegene project (leucegene.ca). Reads were mapped to the reference genome hg38 using STAR v2.7.1 (11). Gene and transcript expression levels were quantified with RSEM version 1.3.2 (12). Transcripts per million (TPM) were calculated for each transcript by normalizing for transcript effective length and library size. Clinical data including age, gender, cytogenetic risk, *FLT3*-ITD and NPM1 mutation status and WBC count at diagnosis was collected and validated by the Quebec leukemia cell bank. All patients from this cohort provided signed informed consent and this study was approved by the local research ethics board. The clinical and gene expression data for the genes of interest in the ADE-RS5 and pLSC6 scores from this cohort were provided by investigators of the Leucegene group.

All the gene expression data was log2 transformed before subsequent statistical analysis. One was added to the normalized RNA-Seq expression data before its log2 transformation. Supplementary Table 2 list 11 probes used for validation of LSC6 and ADE-RS scores using data from Illumina HumanHT-12 V4.0 expression beadchip, U133A and the TaqMan assays.

The R script codes are available used for development of ADE-RS is available at GitHub (https://github.com/Abdelrahman-Elsayed/kit-nfold-cv-glmnet/blob/master/kit-nfold-cv-glmnet-v0.R) and step by step flow chart is shown below.

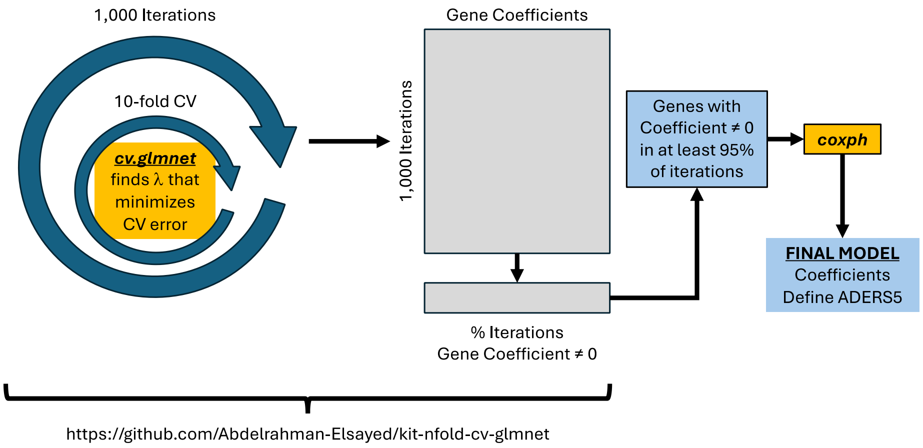

Supplementary Table 1. Sixty-seven Genes with pharmacological significance to ara-C, daunorubicin and etoposide (ADE) included in the study with details on its biological relevance to ADE.

**Sources of drug resistance**	**Genes involved in cytarabine (ara-C) metabolism**	**Genes involved in daunorubicin (DNR) metabolism**	**Genes involved in etoposide metabolism**
1- Reduction of drug uptake into the tumor cell due to impaired function of solute carrier (SLC) transporters	**SLC29A1** is responsible for about 80% of ara-C influx inside leukemic cells(13), in addition to **SLC28A1** and **SLC28A3** with a minor role(14). **SLC22A4** also found as an ara-C high affinity carrier.(15)	**SLC22A1** and **SLCO1B1** were shown to be a high-affinity transporter for daunorubicin.(16)	**SLC22A5** is shown to be an influx trans-porter for etoposide.(17)
2- Increased ability of the tumor cell to pump out pharmacologically active agents through ATP-binding cassette (ABC) efflux transporters	A study demonstrated that higher expression of the efflux transporter **ABCC10** reduced the accumulation of ara-C.(18)	**ABCB1** encoded by the multidrug resistance (MDR1) gene decreases the intracellular content of daunorubicin. In addition, **ABCC1, ABCC2, ABCC3, ABCG2, ABCA3** and **RALBP1** also reported to act as DNR efflux transporters.(19, 20)	**ABCB1** act as an efflux transporter for etoposide. In addition, **ABCC1, ABCC2** and **ABCC3** were also reported to act as etoposide efflux transporters.(21)
3- Decrease in the expression or activity of enzymes responsible for the activation of pro-drugs	**DCK** is the rate-limiting enzyme that catalyzes the first step in the activation of ara-C, converting ara-C to ara-CMP.(22) **CMPK1** Catalyze the second step of cytarabine activation to ara-CDP.(23) Diphosphate kinases including **NME1, NME2** and **NME3** catalyze the final step of ara-C activation to ara-CTP	NADH dehydrogenases including **NDUFS2, NDUFS3** and **NDUFS7** catalyze conversion of anthracyclines to its semiquinone metabolites, which are more cytotoxic than the parental molecules.(24) **XDH, NQO1** and **NOS** enzymes were also reported previously to be involved in anthracyclines semiquinone formation.(25)	**CYP3A4, CYP3A5** are Involved in the conversion of etoposide to its catechol metabolites.(26) Myeloperoxidase (**MPO**) further oxidize catechol to quinone. These metabolites are more oxidatively active.(27)
4- Increase in the expression or activity of enzymes responsible for the drug inactivation	**CDA** play a major role in ara-C inactivation by irreversibly deaminating ara-C to ara-U.(28) **NT5C2** and **NT5C3A** dephosphorylate ara-CMP back to ara-C. In addition, **DCTD**, **CTPs** enzymes, **RRM**s and **SAMHD1** play an important role in cytarabine deactivation.(29, 30)	Carbonyl reductases such as **CBR1** and **CBR3** convert daunoruicin to its less active metabolite daunorubicinol.(31) Aldo-keto reductases such as **AKR1A1, AKR1C1 and AKR1C3** play the same role in the acquisition of resistance.(32) **GPX1** and **SOD1** deactivate DNR associated ROS.(33)	**GSTT1** and **GSTP1** are involved in glutathione conjugation to inactivate etoposide and its metabolites.(34) In addition, **UGT1A1** is involved in etoposide glucurodination and inactivation.(35)
5- Altered expression or function of the molecular targets of the drugs	**POLA1** and **POLA2** encode for catalytic and accessory subunits of DNA polymerase alpha, essential for DNA replication.(36)	**TOP2A** and **TOP2B** are responsible for relegation of DNA breaks and considered as daunorubicin targets.(37)	**TOP2A** and **TOP2B** are also considered as targets for etoposide.(38)

Supplementary Table 2. Distribution of patient characteristics in discovery AML02cohort by pLSC6, ADE-RS5, and integrated LSC6/ASERS5 score groups.

		pLSC6 Score Groups			ADE-RS5 Score Groups			Integrated LSC6/ADERS5 Four Score Groups				
**Variables**	**Level**	**Low**	**High**	**Pvalue**	**Low**	**High**	**Pvalue**	**Group 1**	**Group 2**	**Group 3**	**Group 4**	**Pvalue**
**Treatemtn Arm**	**HDAC**	47(49)	27(41.5)	0.418	48(49.5)	26(40.6)	0.3382	37(51.4)	10(41.7)	11(44)	16(40)	0.6586
	**LDAC**	49(51)	38(58.5)		49(50.5)	38(59.4)		35(48.6)	14(58.3)	14(56)	24(60)	
**Gender**	**Female**	45(46.4)	29(43.9)	0.8698	47(48)	27(41.5)	0.426	37(50.7)	8(33.3)	10(40)	19(46.3)	0.4909
	**Male**	52(53.6)	37(56.1)		51(52)	38(58.5)		36(49.3)	16(66.7)	15(60)	22(53.7)	
**Age group**	**<10**	47(48.5)	36(54.5)	0.5186	48(49)	35(53.8)	0.6342	38(52.1)	9(37.5)	10(40)	26(63.4)	0.1391
	**>=10**	50(51.5)	30(45.5)		50(51)	30(46.2)		35(47.9)	15(62.5)	15(60)	15(36.6)	
**Race**	**White**	69(71.9)	46(69.7)	0.962	66(68)	49(75.4)	0.5854	51(70.8)	18(75)	15(60)	31(75.6)	0.8986
	**Black**	18(18.8)	13(19.7)		20(20.6)	11(16.9)		14(19.4)	4(16.7)	6(24)	7(17.1)	
	**Other**	9(9.4)	7(10.6)		11(11.3)	5(7.7)		7(9.7)	2(8.3)	4(16)	3(7.3)	
**Risk group**	**Low**	51(52.6)	4(6.1)	**<0.0001**	39(39.8)	16(24.6)	0.0845	38(52.1)	13(54.2)	1(4)	3(7.3)	**<0.0001**
	**Standard**	38(39.2)	27(40.9)		38(38.8)	27(41.5)		28(38.4)	10(41.7)	10(40)	17(41.5)	
	**High**	8(8.2)	35(53)		21(21.4)	22(33.8)		7(9.6)	1(4.2)	14(56)	21(51.2)	
**Cytogenetic group**	**t(8;21)**	24(25)	0(0)	**<0.0001**	15(15.5)	9(14.1)	**0.0423**	15(20.8)	9(37.5)	0(0)	0(0)	**<0.0001**
	**inv(16)**	21(21.9)	0(0)		18(18.6)	3(4.7)		18(25)	3(12.5)	0(0)	0(0)	
	**11q23**	22(22.9)	10(15.4)		21(21.6)	11(17.2)		19(26.4)	3(12.5)	2(8)	8(20)	
	**Normal**	16(16.7)	27(41.5)		24(24.7)	19(29.7)		12(16.7)	4(16.7)	12(48)	15(37.5)	
	**Other**	13(13.5)	28(43.1)		19(19.6)	22(34.4)		8(11.1)	5(20.8)	11(44)	17(42.5)	
**FLT3 status**	**Wild type**	87(90.6)	42(63.6)	**<0.0001**	81(83.5)	48(73.8)	0.1587	65(90.3)	22(91.7)	16(64)	26(63.4)	**0.0008**
	**ITD/Mutation**	9(9.4)	24(36.4)		16(16.5)	17(26.2)		7(9.7)	2(8.3)	9(36)	15(36.6)	
**WBC group**	**<50**	68(70.1)	44(66.7)	0.7221	68(69.4)	44(67.7)	0.8695	52(71.2)	16(66.7)	16(64)	28(68.3)	0.9236
	**>=50**	29(29.9)	22(33.3)		30(30.6)	21(32.3)		21(28.8)	8(33.3)	9(36)	13(31.7)	
**Bone marrow blast(%)**		57.87(23.79)	60.6(25.54)	0.4162	53.91(25.02)	66.85(21.56)	**0.0012**	55.19(24.07)	66.42(21.2)	50.33(27.72)	67.09(22.04)	**0.0124**
**Induction I response**	**CR**	88(90.7)	41(62.1)	**0.0002**	82(83.7)	47(72.3)	0.0981	67(91.8)	21(87.5)	15(60)	26(63.4)	**0.0012**
	**PR**	5(5.2)	10(15.2)		9(9.2)	6(9.2)		4(5.5)	1(4.2)	5(20)	5(12.2)	
	**NR**	4(4.1)	15(22.7)		7(7.1)	12(18.5)		2(2.7)	2(8.3)	5(20)	10(24.4)	
**Induction II response**	**CR**	95(99)	56(88.9)	**0.0061**	95(97.9)	56(90.3)	0.0684	71(98.6)	24(100)	24(96)	32(84.2)	**0.025**
	**PR**	1(1)	6(9.5)		2(2.1)	5(8.1)		1(1.4)	0(0)	1(4)	5(13.2)	
	**NR**	0(0)	1(1.6)		0(0)	1(1.6)		0(0)	0(0)	0(0)	1(2.6)	
**Induction I MRD**	**Negative**	73(81.1)	20(30.8)	**<0.0001**	63(68.5)	30(47.6)	**0.0133**	54(80.6)	19(82.6)	9(36)	11(27.5)	**<0.0001**
	**Positive**	17(18.9)	45(69.2)		29(31.5)	33(52.4)		13(19.4)	4(17.4)	16(64)	29(72.5)	
**Induction II MRD**	**Negative**	82(95.3)	33(53.2)	**<0.0001**	74(82.2)	41(70.7)	0.1057	61(93.8)	21(100)	13(52)	20(54.1)	**<0.0001**
	**Positive**	4(4.7)	29(46.8)		16(17.8)	17(29.3)		4(6.2)	0(0)	12(48)	17(45.9)	
**Event-free Survial (5-years)**		78.27(4.2)	34.53(5.89)	**<0.0001**	77.49(4.23)	34.52(6.01)	**<0.0001**	86.22(4.05)	54.17(10.17)	52(9.99)	23.78(6.74)	**<0.0001**
**Overall Survial (5-years)**		87.48(3.38)	46.45(6.2)	**<0.0001**	85.66(3.55)	47.71(6.34)	**<0.0001**	93.13(2.97)	69.7(9.57)	64(9.6)	36.1(7.58)	**<0.0001**

Supplementary Table 3. U133 microarray GenChip, Illumina HumanHT-12 V4.0 expression bead chip probe IDs and RT-PCR assay IDs for 11 genes represented on pLSC6 and ADE-RS score equations

Gene name	Signature	RT-PCR probe ID	U133 Microarray Probe-ID	Illumina
DNMT3B	pLSC6	Hs00171876_m1	220668_s_at	ILMN_2328972
GPR56	pLSC6	Hs00173754_m1	212070_at	ILMN_2384122
CD34	pLSC6	Hs02576480_m1	209543_s_at	ILMN_1732799
SPINK2	pLSC6	Hs00221653_m1	206310_at	ILMN_1763516
SOCS2	pLSC6	Hs00919620_m1	203373_at	ILMN_1798926
FAM30A	pLSC6	Hs00796164_s1	220377_at	ILMN_3187535
ABCC1	ADE-RS	Hs01561483_m1	202804_at	ILMN_1802404
CBR1	ADE-RS	Hs00156323_m1	209213_at	ILMN_1809003
DCTD	ADE-RS	Hs01126095_m1	210137_s_at	ILMN_1802456
MPO	ADE-RS	Hs00165162_m1	203948_s_at	ILMN_1705183
TOP2A	ADE-RS	Hs01032137_m1	201292_at	ILMN_1686097
GAPDH		Hs99999905_m1		
ACTB		Hs01060665_g1		

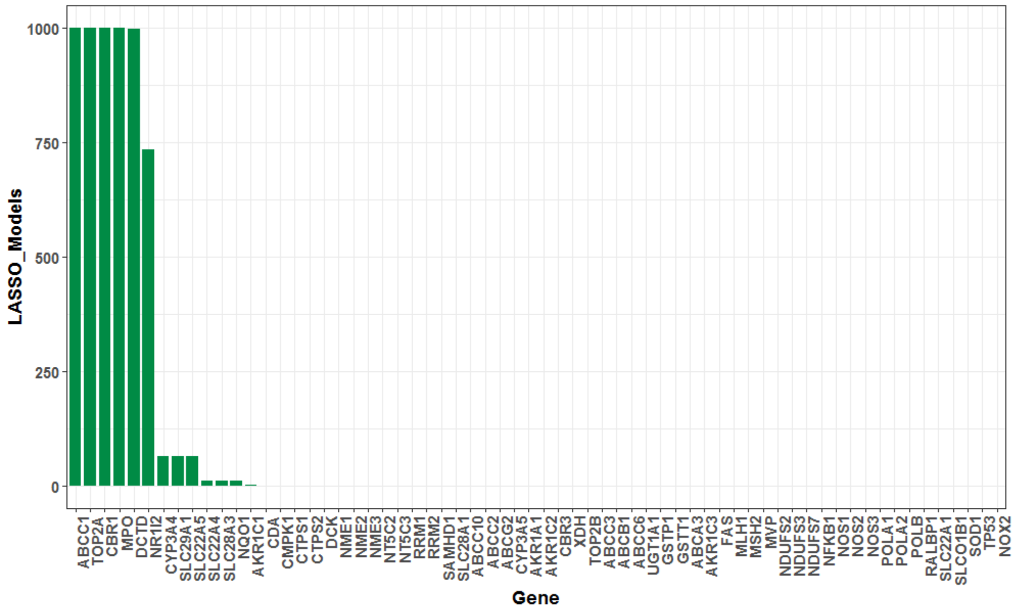

Supplementary Figure 1. Barplot depicting frequency of representation of the genes in 1000 boot-strapping models run using LASSO cox-regression model. **DCTD:** deaminase involved in ara-C inactivation; **CBR1:** carbonyl reductase involved in inactivation of daunorubicin; **MPO:** myeloperoxidase; **ABCC1:** efflux transporter of daunorubicin and etoposide; **TOP2A:** DNA topoisomerase II alpha, target for daunorubicin and etoposide

A) ADERS score and EFS within pLSC6 groups

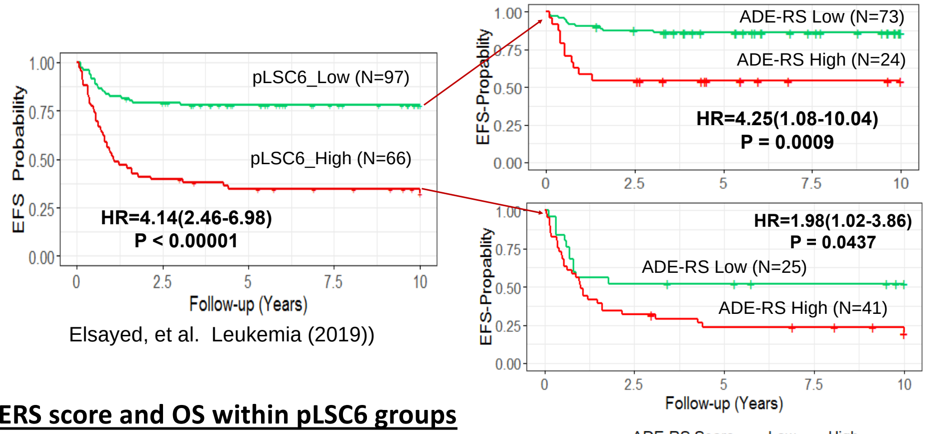

B) ADERS score and OS within pLSC6 groups

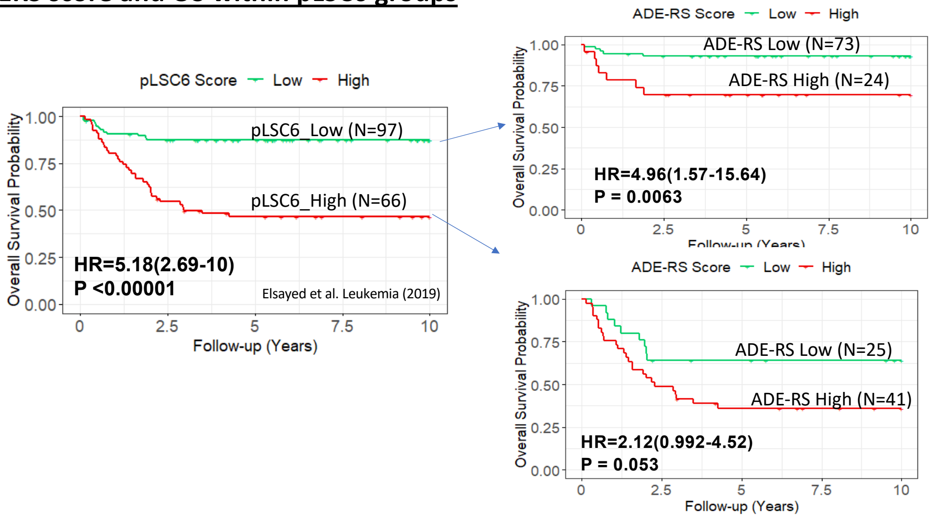

Supplementary Figure 2. EFS (A) and OS (B) survival curves of ADE-RS scores within pLSC6 score groups (high Vs low; defined Elsayed et al, 2020).

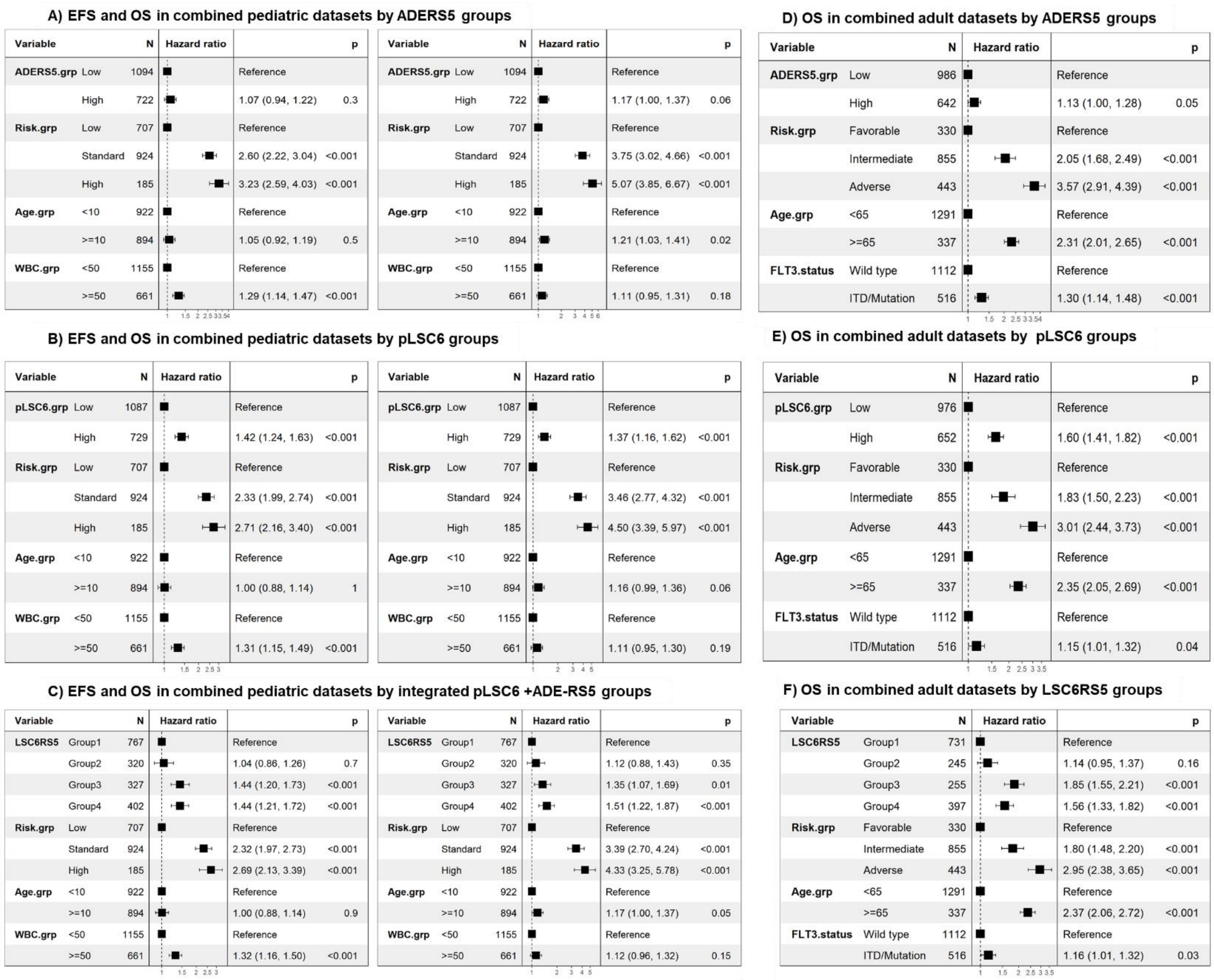

Supplementary Figure 3: Forest plots showing results of multivariable cox regression analysis of association of ADE-RS5, pLSC6 and the integrated LSC6/ADE-RS5 score groups with OS and EFS in 1,816 pediatric (A, B, C respectively), and association with OS in 1,628 adult AML patients (D, E, and F) from multiple multi-site clinical trials after adjusting for risk group, age, and WBC.

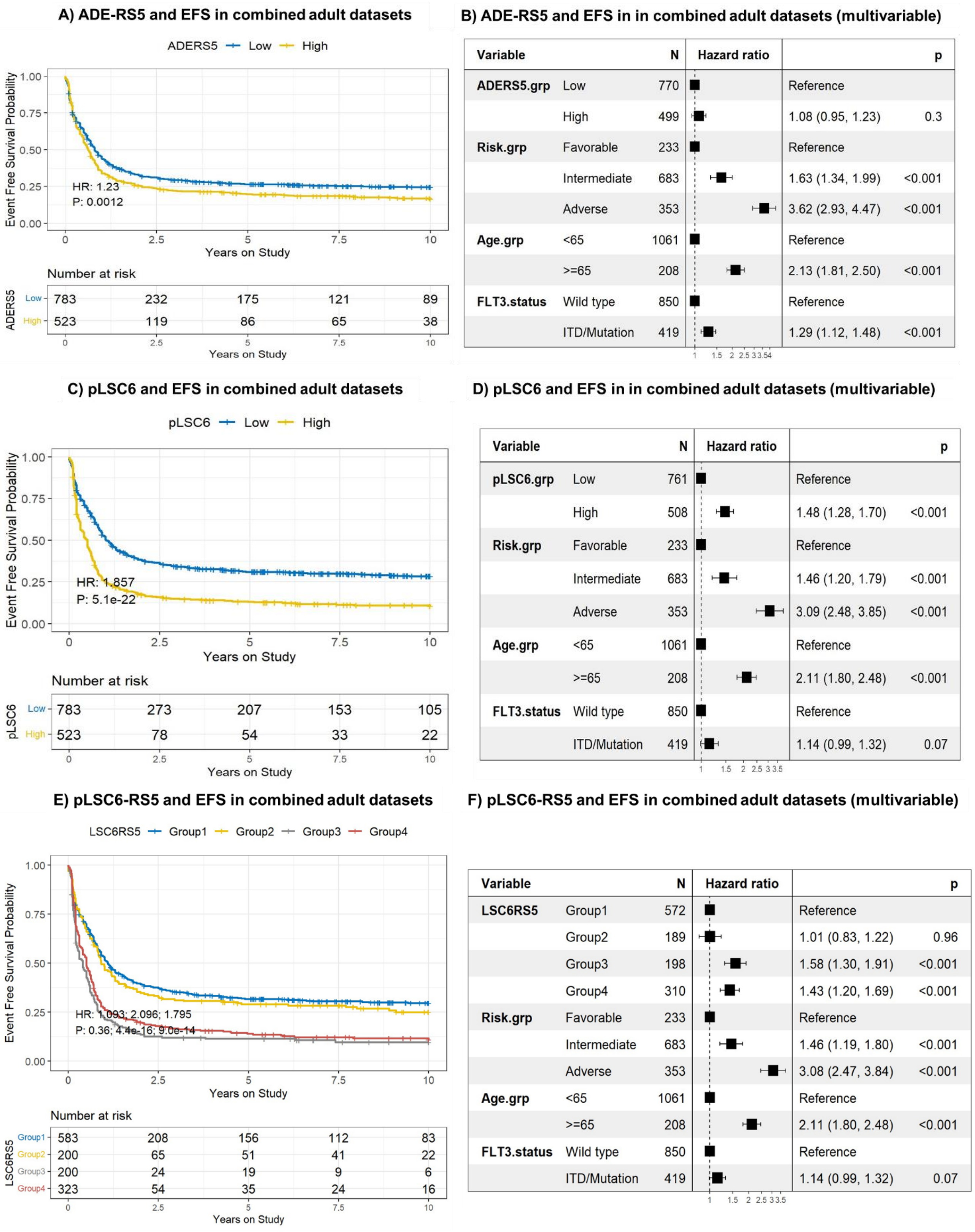

Supplementary Figure 4: Association of ADE-RS5 (A), pLSC6 (C) and the integrated score groups (E) with EFS in 1,306 adult AML patients (German_GSE37642, Leucegene and GSE6891). Forest plots showing results of multivariable cox regression analysis of association of ADE-RS5 (B), pLSC6 (D)and the integrated score groups (F) and EFS after adjusting for risk group assignment, FLT3 status and age. * EFS data was not available from GSE68833-TCGA and Beat-AML datasets

A) OS by pLSC6 in combined adult datasets (age<65)

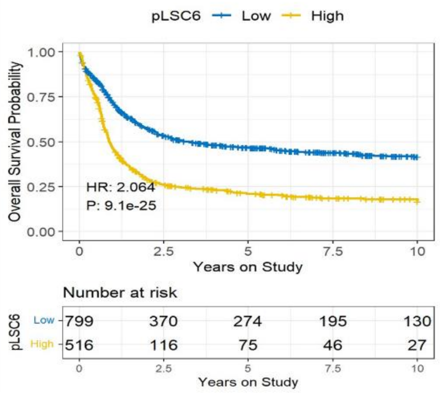

B) OS by pLSC6 in combined adult datasets (age<65; multivariable)

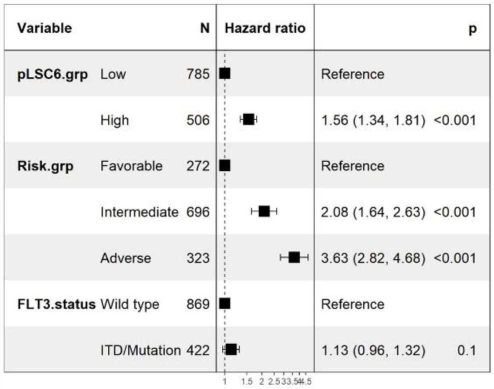

C) OS by pLSC6 in combined adult datasets (age>=65)

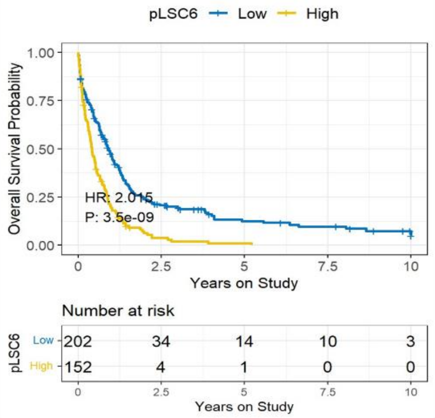

D) OS by pLSC6 in combined adult datasets (age>=65; multivariable)

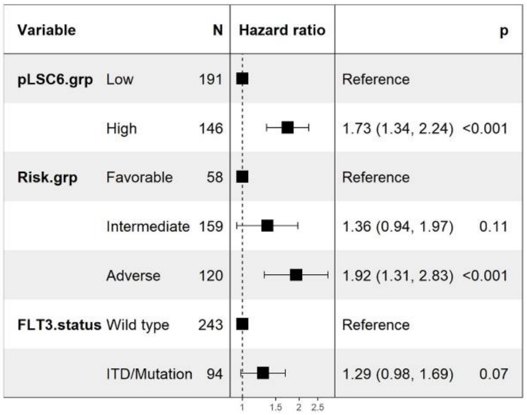

E) OS by ADE-RS5 in combined adult datasets (age<65)

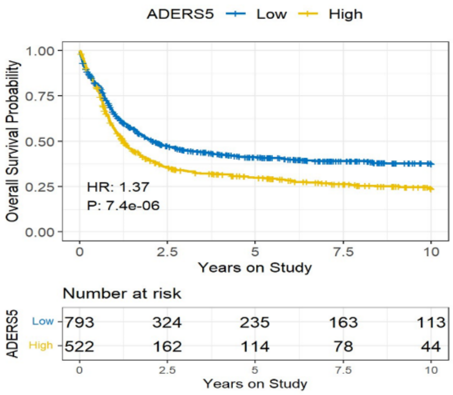

F) OS by ADE-RS5 in combined adult datasets (age<65; multivariable)

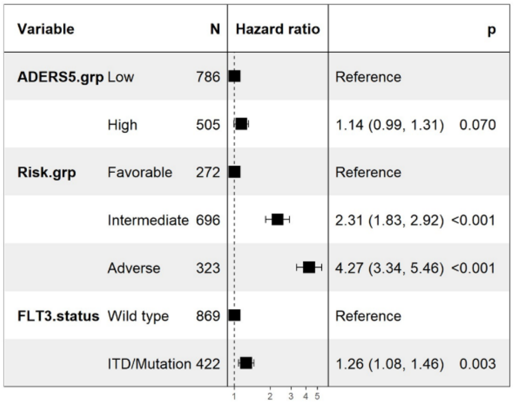

G) OS by ADE-RS5 in combined adult datasets (age>=65)

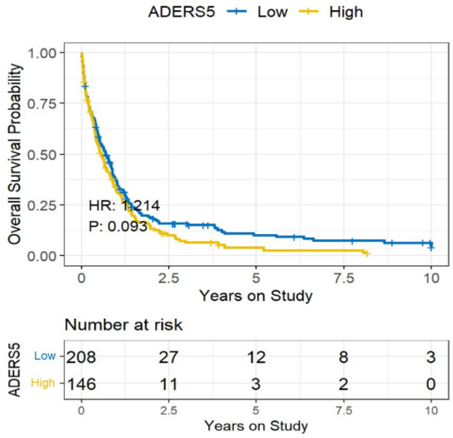

H) OS by ADE-RS5 in combined adult datasets (age>=65; multivariable)

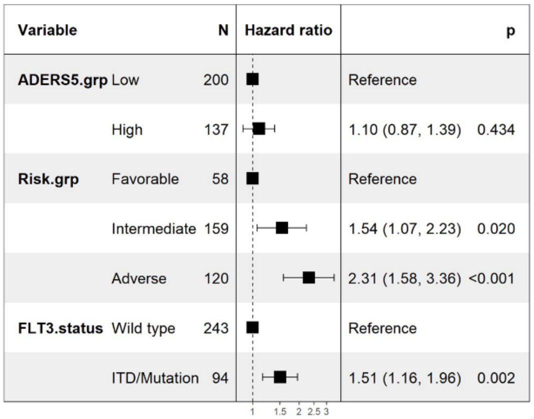

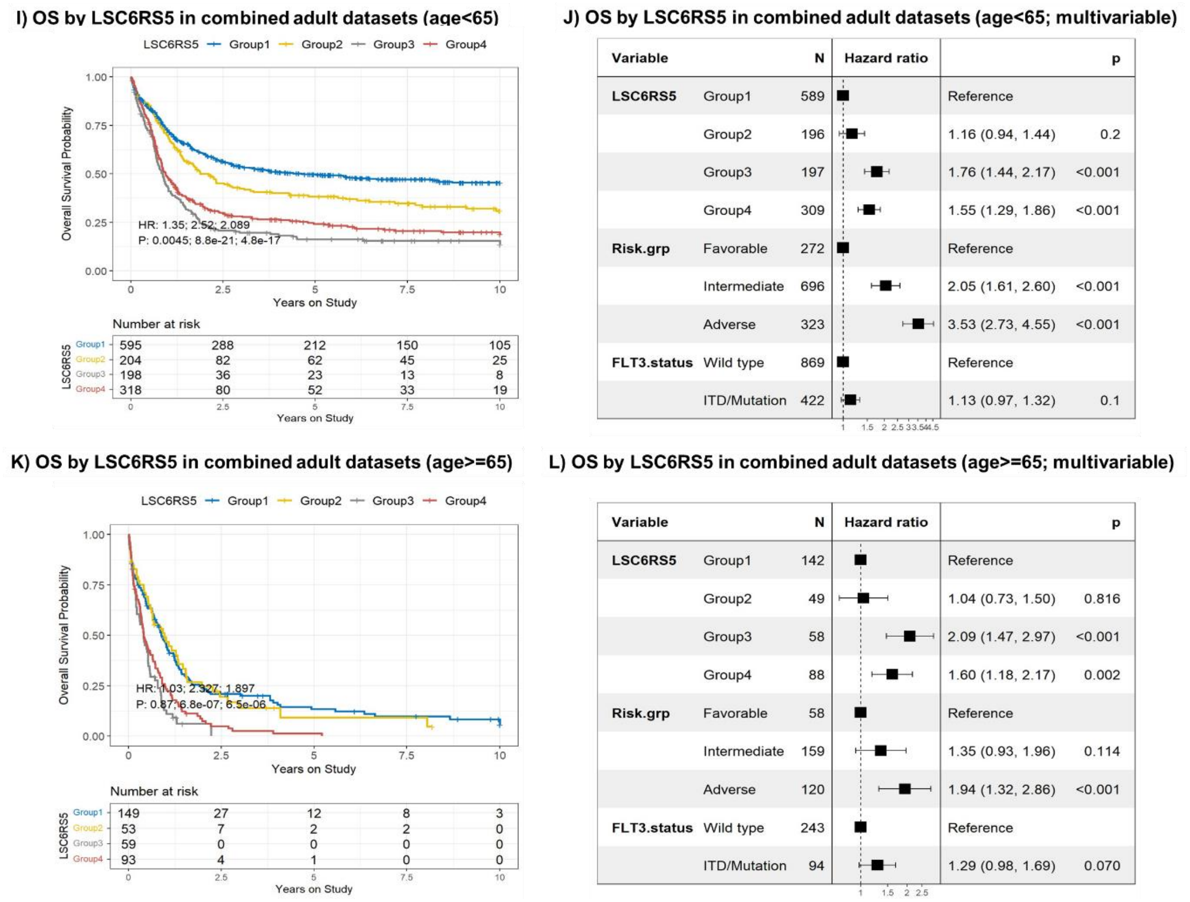

Supplementary Figure 5: Association of ADE-RS, pLSC6 and the integrated LSC6/ADE-RS5 four score groups with OS in 1,669 adult AML patients by age groups (<65 years old, and elderly patients who are >=65 years old).

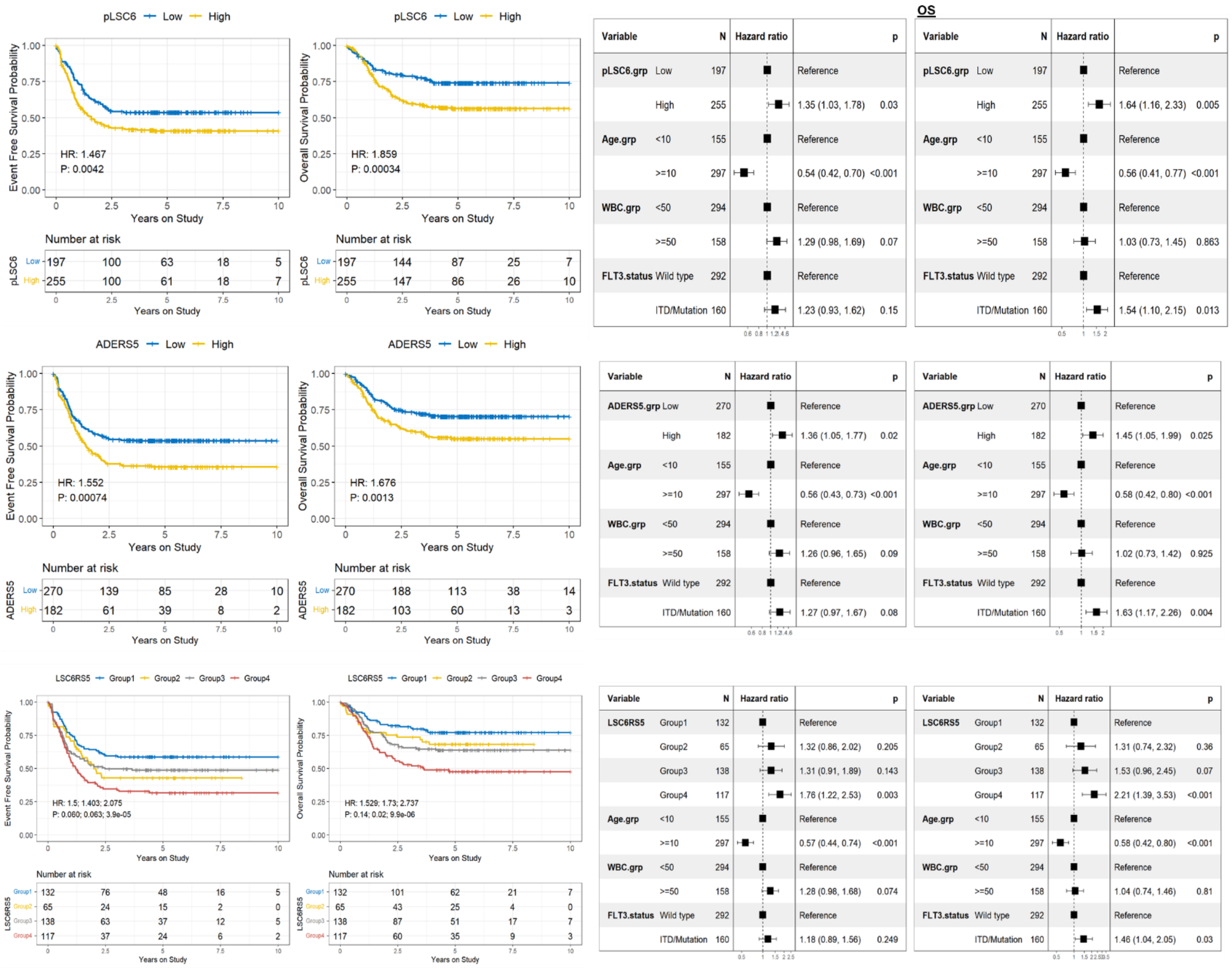
OS
pLSC6 Low High
HR: 1.467
P: 0.0042
HR: 1.859
P: 0.00034
Event Free Survival Probability
Overall Survival Probability
Years on Study
Number at risk
pLSC6 Low 197 100 63 18 5
pLSC6 High 255 100 61 18 7
pLSC6 Low 197 144 87 25 7
pLSC6 High 255 147 86 26 10
Variable N Hazard ratio p
pLSC6.grp Low 197 Reference
High 255 1.35 (1.03, 1.78) 0.03
Age.grp <10 155 Reference
>=10 297 0.54 (0.42, 0.70) <0.001
WBC.grp <50 294 Reference
>=50 158 1.29 (0.98, 1.69) 0.07
FLT3.status Wild type 292 Reference
ITD/Mutation 160 1.23 (0.93, 1.62) 0.15
pLSC6.grp Low 197 Reference
High 255 1.64 (1.16, 2.33) 0.005
Age.grp <10 155 Reference
>=10 297 0.56 (0.41, 0.77) <0.001
WBC.grp <50 294 Reference
>=50 158 1.03 (0.73, 1.45) 0.863
FLT3.status Wild type 292 Reference
ITD/Mutation 160 1.54 (1.10, 2.15) 0.013
ADERS5 Low High
HR: 1.552
P: 0.00074
HR: 1.676
P: 0.0013
ADERS5 Low 270 139 85 28 10
ADERS5 High 182 61 39 8 2
ADERS5 Low 270 188 113 38 14
ADERS5 High 182 103 60 13 3
ADERS5.grp Low 270 Reference
High 182 1.36 (1.05, 1.77) 0.02
Age.grp <10 155 Reference
>=10 297 0.56 (0.43, 0.73) <0.001
WBC.grp <50 294 Reference
>=50 158 1.26 (0.96, 1.65) 0.09
FLT3.status Wild type 292 Reference
ITD/Mutation 160 1.27 (0.97, 1.67) 0.08
ADERS5.grp Low 270 Reference
High 182 1.45 (1.05, 1.99) 0.025
Age.grp <10 155 Reference
>=10 297 0.58 (0.42, 0.80) <0.001
WBC.grp <50 294 Reference
>=50 158 1.02 (0.73, 1.42) 0.925
FLT3.status Wild type 292 Reference
ITD/Mutation 160 1.63 (1.17, 2.26) 0.004
LSC6RS5 Group1 Group2 Group3 Group4
HR: 1.5; 1.403; 2.075
P: 0.060; 0.063; 3.9e-05
HR: 1.529; 1.73; 2.737
P: 0.14; 0.02; 9.9e-06
LSC6RS5 Group1 132 76 48 16 5
Group2 65 24 15 2 0
Group3 138 63 37 12 5
Group4 117 37 24 6 2
LSC6RS5 Group1 132 101 62 21 7
Group2 65 43 25 4 0
Group3 138 87 51 17 7
Group4 117 60 35 9 3
LSC6RS5 Group1 132 Reference
Group2 65 1.32 (0.86, 2.02) 0.205
Group3 138 1.31 (0.91, 1.89) 0.143
Group4 117 1.76 (1.22, 2.53) 0.003
Age.grp <10 155 Reference
>=10 297 0.57 (0.44, 0.74) <0.001
WBC.grp <50 294 Reference
>=50 158 1.28 (0.98, 1.68) 0.074
FLT3.status Wild type 292 Reference
ITD/Mutation 160 1.18 (0.89, 1.56) 0.249
LSC6RS5 Group1 132 Reference
Group2 65 1.31 (0.74, 2.32) 0.36
Group3 138 1.53 (0.96, 2.45) 0.07
Group4 117 2.21 (1.39, 3.53) <0.001
Age.grp <10 155 Reference
>=10 297 0.58 (0.42, 0.80) <0.001
WBC.grp <50 294 Reference
>=50 158 1.04 (0.74, 1.46) 0.81
FLT3.status Wild type 292 Reference
ITD/Mutation 160 1.46 (1.04, 2.05) 0.03

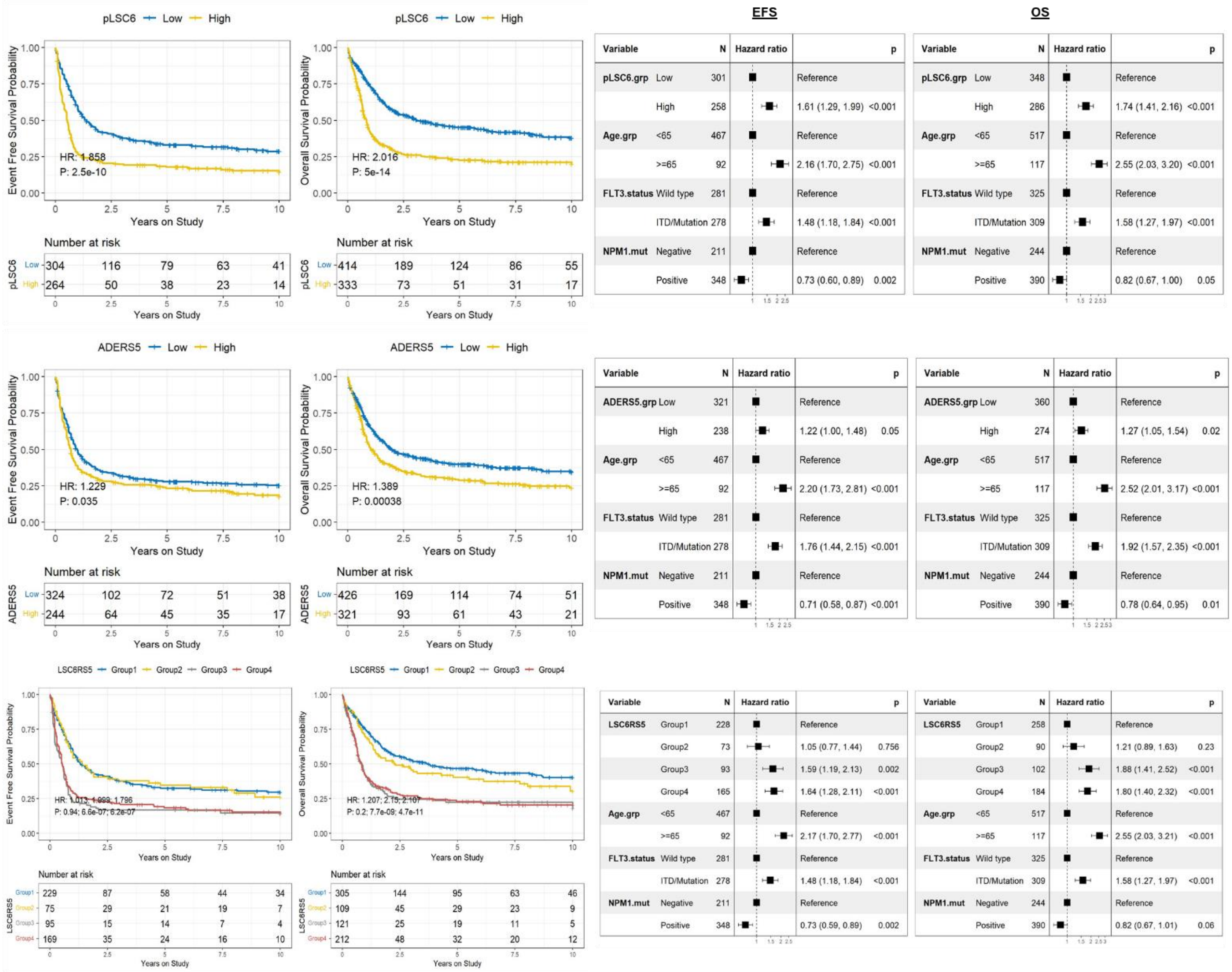

Supplementary Figure 6A. pLSC6, ADERS and integrated score groups in combined pediatric datasets (cytogenetically normal; 452 patients) 6B. LSC6, ADERS and integrated score groups in combined adult datasets (cytogenetically normal; 747 patients)

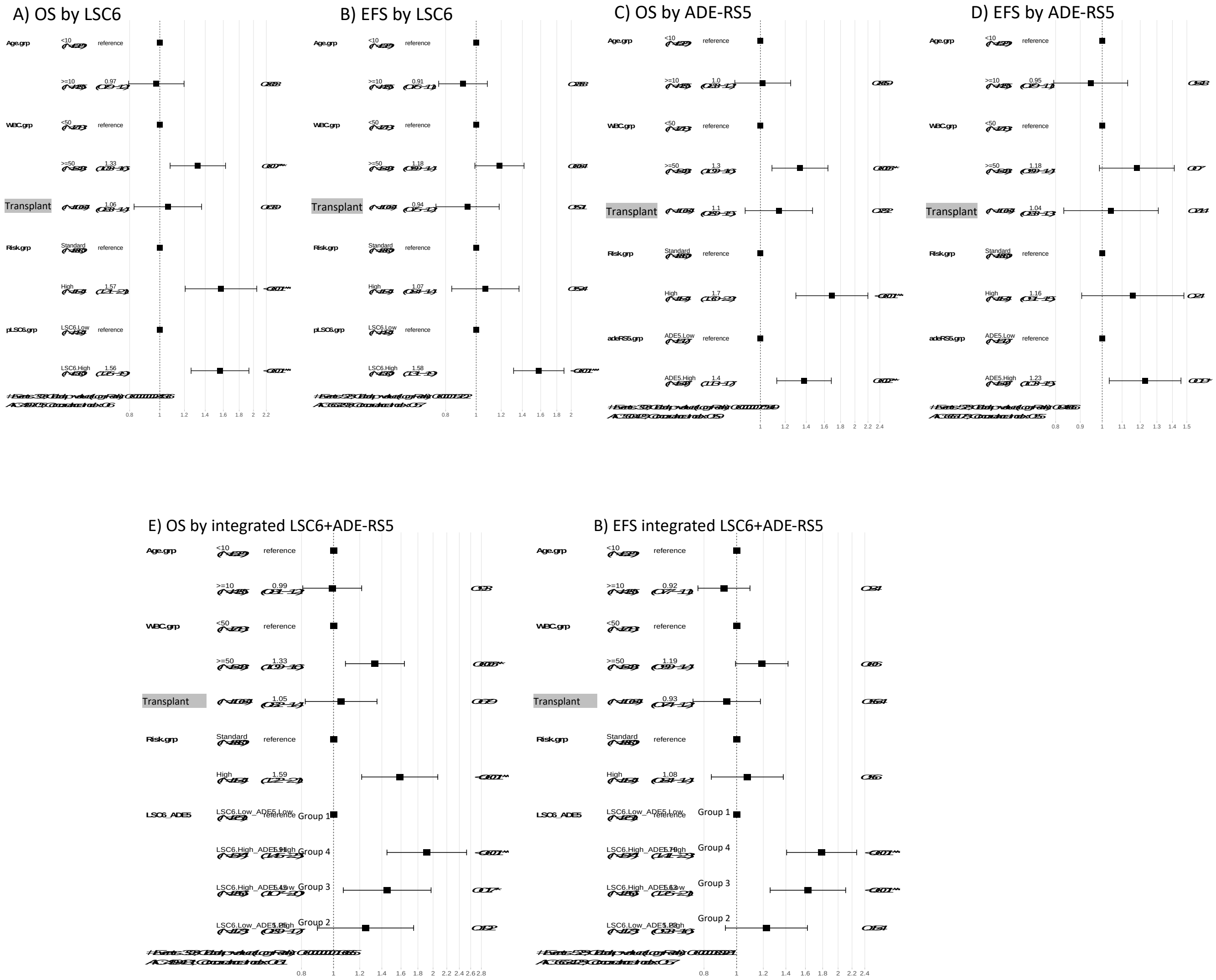

Supplementary Figure 7. Forest plots showing results of multivariable cox regression analysis of association of ADE-RS5 (B), pLSC6 (D)and the integrated score groups (F) for OS and EFS after adjusting for age, WBC, HSCT and risk group assignment in 1064 patients from 4 pediatric AML cohorts.

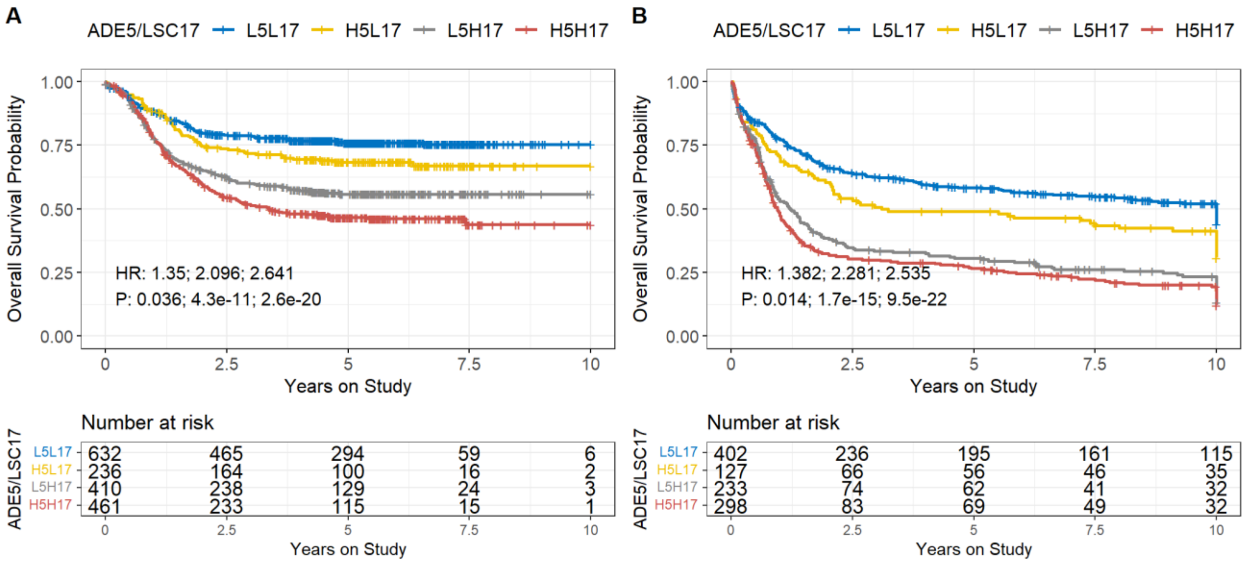

Supplementary Figure 8: Association of integrated score ADE-RS5 and LSC17 with OS in pediatric and adult AML cohorts: L5L17: Low ADE-RS5/Low-LSC17 (Blue); H5L17: High ADE-RS5/Low-LSC17 (Yellow); L5H17: Low ADE-RS5/High-LSC17 (Grey); H5H17: High ADE-RS5/High-LSC17 (Red).

Supplemental References:

1. Gamis AS, Alonzo TA, Meshinchi S, Sung L, Gerbing RB, Raimondi SC, et al. Gemtuzumab ozogamicin in children and adolescents with de novo acute myeloid leukemia improves event-free survival by reducing relapse risk: results from the randomized phase III Children's Oncology Group trial

AAML0531. Journal of clinical oncology : official journal of the American Society of Clinical Oncology. 2014;32(27):3021-32.
2. Cooper TM, Franklin J, Gerbing RB, Alonzo TA, Hurwitz C, Raimondi SC, et al. AAML03P1, a pilot study of the safety of gemtuzumab ozogamicin in combination with chemotherapy for newly diagnosed childhood acute myeloid leukemia: a report from the Children's Oncology Group. Cancer. 2012;118(3):761-9.
3. Aplenc R, Alonzo TA, Gerbing RB, Lange BJ, Hurwitz CA, Wells RJ, et al. Safety and efficacy of gemtuzumab ozogamicin in combination with chemotherapy for pediatric acute myeloid leukemia: a report from the Children's Oncology Group. Journal of clinical oncology : official journal of the American Society of Clinical Oncology. 2008;26(14):2390-3295.
4. Jury EI. Theory and application of the z-transform method: Huntington, N.Y., R.E. Krieger Pub. Co.; 1973.
5. Aplenc R, Meshinchi S, Sung L, Alonzo T, Choi J, Fisher B, et al. Bortezomib with standard chemotherapy for children with acute myeloid leukemia does not improve treatment outcomes: a report from the Children's Oncology Group. Haematologica. 2020;105(7):1879-86.
6. Rubnitz JE, Lacayo NJ, Inaba H, Heym K, Ribeiro RC, Taub J, et al. Clofarabine Can Replace Anthracyclines and Etoposide in Remission Induction Therapy for Childhood Acute Myeloid Leukemia: The AML08 Multicenter, Randomized Phase III Trial. Journal of clinical oncology : official journal of the American Society of Clinical Oncology. 2019;37(23):2072-81.
7. Ley TJ, Miller C, Ding L, Raphael BJ, Mungall AJ, Robertson A, et al. Genomic and epigenomic landscapes of adult de novo acute myeloid leukemia. N Engl J Med. 2013;368(22):2059-74.
8. Buchner T, Berdel WE, Schoch C, Haferlach T, Serve HL, Kienast J, et al. Double induction containing either two courses or one course of high-dose cytarabine plus mitoxantrone and postremission therapy by either autologous stem-cell transplantation or by prolonged maintenance for acute myeloid leukemia. J Clin Oncol. 2006;24(16):2480-9.
9. Herold T, Metzeler KH, Vosberg S, Hartmann L, Rollig C, Stolzel F, et al. Isolated trisomy 13 defines a homogeneous AML subgroup with high frequency of mutations in spliceosome genes and poor prognosis. Blood. 2014;124(8):1304-11.
10. Chuang MK, Chiu YC, Chou WC, Hou HA, Tseng MH, Kuo YY, et al. An mRNA expression signature for prognostication in de novo acute myeloid leukemia patients with normal karyotype. Oncotarget. 2015;6(36):39098-110.
11. Dobin A, Davis CA, Schlesinger F, Drenkow J, Zaleski C, Jha S, et al. STAR: ultrafast universal RNA-seq aligner. Bioinformatics. 2013;29(1):15-21.
12. Li B, Dewey CN. RSEM: accurate transcript quantification from RNA-Seq data with or without a reference genome. BMC Bioinformatics. 2011;12:323.
13. Hubeek I, Stam RW, Peters GJ, Broekhuizen R, Meijerink JP, van Wering ER, et al. The human equilibrative nucleoside transporter 1 mediates in vitro cytarabine sensitivity in childhood acute myeloid leukaemia. Br J Cancer. 2005;93(12):1388-94.
14. Gray JH, Owen RP, Giacomini KM. The concentrative nucleoside transporter family, SLC28. Pflugers Arch. 2004;447(5):728-34.
15. Drenberg CD, Gibson AA, Pounds SB, Shi L, Rhinehart DP, Li L, et al. OCTN1 Is a High-Affinity Carrier of Nucleoside Analogues. Cancer Res. 2017;77(8):2102-11.
16. Drenberg CD, Paugh SW, Pounds SB, Shi L, Orwick SJ, Li L, et al. Inherited variation in OATP1B1 is associated with treatment outcome in acute myeloid leukemia. Clin Pharmacol Ther. 2016;99(6):651-60.
17. Hu C, Lancaster CS, Zuo Z, Hu S, Chen Z, Rubnitz JE, et al. Inhibition of OCTN2-mediated transport of carnitine by etoposide. Mol Cancer Ther. 2012;11(4):921-9.
18. Hopper-Borge E, Xu X, Shen T, Shi Z, Chen ZS, Kruh GD. Human multidrug resistance protein 7 (ABCC10) is a resistance factor for nucleoside analogues and epothilone B. Cancer Res. 2009;69(1):178-84.

19. Ross DD, Doyle LA, Schiffer CA, Lee EJ, Grant CE, Cole SP, et al. Expression of multidrug resistance-associated protein (MRP) mRNA in blast cells from acute myeloid leukemia (AML) patients. Leukemia. 1996;10(1):48-55.
20. Den Boer ML, Pieters R, Veerman AJ. Mechanisms of cellular anthracycline resistance in childhood acute leukemia. Leukemia. 1998;12(11):1657-70.
21. Lagas JS, Fan L, Wagenaar E, Vlaming ML, van Tellingen O, Beijnen JH, et al. P-glycoprotein (P-gp/Abcb1), Abcc2, and Abcc3 determine the pharmacokinetics of etoposide. Clin Cancer Res. 2010;16(1):130-40.
22. Bhalla K, Nayak R, Grant S. Isolation and characterization of a deoxycytidine kinase-deficient human promyelocytic leukemic cell line highly resistant to 1-beta-D- arabinofuranosylcytosine. Cancer Res. 1984;44(11):5029-37.
23. Liou JY, Dutschman GE, Lam W, Jiang Z, Cheng YC. Characterization of human UMP/CMP kinase and its phosphorylation of D- and L-form deoxycytidine analogue monophosphates. Cancer Res. 2002;62(6):1624-31.
24. Pawłowska J, Tarasiuk J, Wolf CR, Paine MJ, Borowski E. Differential ability of cytostatics from anthraquinone group to generate free radicals in three enzymatic systems: NADH dehydrogenase, NADPH cytochrome P450 reductase, and xanthine oxidase. Oncol Res. 2003;13(5):245-52.
25. Vásquez-Vivar J, Martasek P, Hogg N, Masters BS, Pritchard KA, Kalyanaraman B. Endothelial nitric oxide synthase-dependent superoxide generation from adriamycin. Biochemistry. 1997;36(38):11293-7.
26. Zhuo X, Zheng N, Felix CA, Blair IA. Kinetics and regulation of cytochrome P450-mediated etoposide metabolism. Drug Metab Dispos. 2004;32(9):993-1000.
27. Kagan VE, Kuzmenko AI, Tyurina YY, Shvedova AA, Matsura T, Yalowich JC. Pro-oxidant and antioxidant mechanisms of etoposide in HL-60 cells: role of myeloperoxidase. Cancer Res. 2001;61(21):7777-84.
28. Schröder JK, Kirch C, Seeber S, Schütte J. Structural and functional analysis of the cytidine deaminase gene in patients with acute myeloid leukaemia. Br J Haematol. 1998;103(4):1096-103.
29. Verschuur AC, Brinkman J, Van Gennip AH, Leen R, Vet RJ, Evers LM, et al. Cyclopentenyl cytosine induces apoptosis and increases cytarabine-induced apoptosis in a T-lymphoblastic leukemic cell-line. Leuk Res. 2001;25(10):891-900.
30. Herold N, Rudd SG, Ljungblad L, Sanjiv K, Myrberg IH, Paulin CB, et al. Targeting SAMHD1 with the Vpx protein to improve cytarabine therapy for hematological malignancies. Nat Med. 2017;23(2):256-63.
31. Varatharajan S, Abraham A, Zhang W, Shaji RV, Ahmed R, George B, et al. Carbonyl reductase 1 expression influences daunorubicin metabolism in acute myeloid leukemia. Eur J Clin Pharmacol. 2012;68(12):1577-86.
32. Matsunaga T, Yamaguchi A, Morikawa Y, Kezuka C, Takazawa H, Endo S, et al. Induction of aldo-keto reductases (AKR1C1 and AKR1C3) abolishes the efficacy of daunorubicin chemotherapy for leukemic U937 cells. Anticancer Drugs. 2014;25(8):868-77.
33. Miyamoto Y, Koh YH, Park YS, Fujiwara N, Sakiyama H, Misonou Y, et al. Oxidative stress caused by inactivation of glutathione peroxidase and adaptive responses. Biol Chem. 2003;384(4):567-74.
34. Mans DR, Lafleur MV, Westmijze EJ, Horn IR, Bets D, Schuurhuis GJ, et al. Reactions of glutathione with the catechol, the ortho-quinone and the semi-quinone free radical of etoposide. Consequences for DNA inactivation. Biochem Pharmacol. 1992;43(8):1761-8.
35. Wen Z, Tallman MN, Ali SY, Smith PC. UDP-glucuronosyltransferase 1A1 is the principal enzyme responsible for etoposide glucuronidation in human liver and intestinal microsomes: structural characterization of phenolic and alcoholic glucuronides of etoposide and estimation of enzyme kinetics. Drug Metab Dispos. 2007;35(3):371-80.

36. Han S, Hickey RJ, Tom TD, Wills PW, Syväoja JE, Malkas LH. Differential inhibition of the human cell DNA replication complex-associated DNA polymerases by the antimetabolite 1-beta-D-arabinofuranosylcytosine triphosphate (ara-CTP). Biochem Pharmacol. 2000;60(3):403-11.
37. Vavrova A, Jansova H, Mackova E, Machacek M, Haskova P, Tichotova L, et al. Catalytic inhibitors of topoisomerase II differently modulate the toxicity of anthracyclines in cardiac and cancer cells. PLoS One. 2013;8(10):e76676.
38. Burden DA, Osheroff N. Mechanism of action of eukaryotic topoisomerase II and drugs targeted to the enzyme. Biochim Biophys Acta. 1998;1400(1-3):139-54.
